# Alterations of bacteriome, mycobiome and metabolome characteristics in PCOS patients with normal/overweight individuals

**DOI:** 10.1186/s13048-022-01051-8

**Published:** 2022-10-28

**Authors:** Guoshu Yin, Fu Chen, Guishan Chen, Xiaoping Yang, Qingxia Huang, Lan Chen, Minjie Chen, Weichun Zhang, Miaoqiong Ou, Man Cao, Hong Lin, Man Chen, Hongzhi Xu, Jianlin Ren, Yongsong Chen, Zhangran Chen

**Affiliations:** 1 Department of Endocrinology, the First Affiliated Hospital of Shantou University Medical College, Shantou, 515041 China; 2 Department of Clinical Nutrition, the First Affiliated Hospital of Shantou University Medical College, Shantou, 515041 China; 3 Department of Endocrinology, Chaoyang Dafeng Hospital, Shantou, 515154 China; 4 Department of Mathematics and Numerical Simulation and High-Performance Computing Laboratory, School of Sciences, Nanchang University, Nanchang, 330031 China; 5 Department of Reproductive Center, the First Affiliated Hospital of Shantou University Medical College, Shantou, 515041 China; 6 Institute for Microbial Ecology, School of Medicine, Xiamen University, Xiamen, 361005 China

**Keywords:** PCOS, Mycobiome, Diagnostic model, Metabolome, Obesity

## Abstract

To characterize the gut bacteriome, mycobiome and serum metabolome profiles in polycystic ovary syndrome (PCOS) patients with normal/overweight individuals and evaluate a potential microbiota-related diagnostic method development for PCOS, 16S rRNA and ITS2 gene sequencing using 88 fecal samples and 87 metabolome analysis from serum samples are conducted and PCOS classifiers based on multiomics markers are constructed. There are significant bacterial, fungal community and metabolite differences among PCOS patients and healthy volunteers with normal/overweight individuals. Healthy individuals with overweight/obesity display less abnormal metabolism than PCOS patients and uniquely higher abundance of the fungal genus *Mortierella*. Nine bacterial genera, 4 predicted pathways, 11 fungal genera and top 30 metabolites are screened out which distinguish PCOS from healthy controls, with AUCs of 0.84, 0.64, 0.85 and 1, respectively. The metabolite-derived model is more accurate than the microbe-based model in discriminating normal BMI PCOS (PCOS-LB) from normal BMI healthy (Healthy-LB), PCOS-HB from Healthy-HB. Featured bacteria, fungi, predicted pathways and serum metabolites display higher associations with free androgen index (FAI) in the cooccurrence network. In conclusion, our data reveal that hyperandrogenemia plays a central role in the dysbiosis of intestinal microecology and the change in metabolic status in patients with PCOS and that its effect exceeds the role of BMI. Healthy women with high BMI showed unique microbiota and metabolic features.The priority of predictive models in discriminating PCOS from healthy status in this study were serum metabolites, fungal taxa and bacterial taxa.

**Supplementary Information:**

The online version contains supplementary material available at 10.1186/s13048-022-01051-8.

## Introduction

Polycystic ovary syndrome (PCOS) is the most common endocrine disorder that causes infertility in reproductive-aged women [1], which is also associated with insulin resistance (IR), type 2 diabetes mellitus (T2DM), dyslipidemia, nonalcoholic fatty liver disease (NAFLD) and cardiovascular disease [2, 3]. To date, the precise underlying triggers for PCOS remain unclear, but multifactorial factors, including genetics, intrauterine environment, lifestyle and gut microbiota, are thought to be involved in its development.

Microbiota dysbiosis is associated with endocrine and metabolic diseases such as obesity [4], diabetes [5] and PCOS [6, 7]. Recent studies have shown that gut microbiota of PCOS patients differs from that of healthy controls. Qi et al. [7] found that *Bacteroides vulgatus* was markedly elevated in PCOS individuals. Yang et al. [8] showed that *Bacteroides* is a key microbial biomarker for PCOS and even has diagnostic value. The dynamic change of predominant bacteria in PCOS may be affected by race, lifestyle [9], disease severity and the sample size. Previously, we found that *Prevotella*_9, *Dorea*, *Maihella* and *Slackia* were significantly changed in PCOS patients [6]. Nevertheless, whether the gut microbiota contributes to the occurrence and development of PCOS needs further investigation.

Recent evidence suggests that fungi influence local and peripheral immune responses, enhance relevant disease status [10], trigger the occurrence and development of diseases, such as colitis [11], colorectal cancer [12], primary sclerosing cholangitis [13], alcohol-associated liver disease [14] and COVID-19 [15]. Although limited reports have indicated that fungi play a certain role in endocrine and metabolic diseases, the illumination of their role is still in its infancy. Mar Rodríguez et al. [16] firstly revealed gut fungal composition alterations in obese patients. Honkanen et al. [17] showed that bacterial and fungal dysbiosis may be associated with the development of type 1 diabetes mellitus (T1D) in children with beta-cell autoimmunity. Patients with coronary heart disease complicated with NAFLD show an increase in the abundance of *Preussia*, *Xylodon* and *Cladorrhinum* and a reduction of *Candida glabrata* and *Ganoderma* [18].

Metabolomics is a useful method to discover and identify metabolites involved in disease [19, 20]. Our previous study showed that *Prevotella*_9 correlates positively with Lysopc 18:1, Glu-Gln, lysophosphatidyl choline (LPC) 22:1, PC(14:1E/8:0), and LPC 17:2 [6] but negatively with estrone sulfate [6]. The correlation between the gut microbiota and plasma metabolites suggests that the former may participate in the metabolic pathway of PCOS. Therefore, integrated analysis of multiomics data from the gut bacteriome, mycobiome, metabolome and phenome may provide a clue to mechanistic links between PCOS and the gut microbiota. Furthermore, metabolomics provides potential metabolic markers for the prognosis and diagnosis of PCOS [19, 20]. Daan et al. reported that retinol-binding protein 4 (RBP-4), dipeptidyl peptidase IV (DPP-IV) and adiponectin are potential discriminative markers for PCOS with obvious hyperandrogenemia [21].

PCOS may have different etiological causes, and currently we can see that PCOS patients with normal/overweight individuals also differ in clinical phenotypes, such as levels of insulin and polyunsaturated fatty acids including arachidonic acid [22]. Dapas et al. [23] showed that reproductive and metabolic subtypes of PCOS appeared to have distinct genetic architecture and these two subtypes had obvious difference of BMI. Therefore, we are of great concern about the etiological differences in obese and lean PCOS patients and the differences in their gut microbes and plasma metabolites.

In this study, PCOS patients and healthy volunteers with different body mass index (BMI) levels were used to determine gut bacteria and fungi by 16S and ITS2 gene sequencing methods, and serum metabolites from widely targeted metabolomes. The microbiome and metabolome data were then integrated to distinguish PCOS-related alterations in fecal microbial and serum metabolic features, and potential links and roles of the microbiome and metabolome in disease diagnosis were assessed.

## Methods

### Subject recruitment

The study was a cross-sectional study and all experimental procedures were approved by the Ethics Committee of the First Affiliated Hospital Shantou University Medical College. The biological sample banks, including plasma, DNA and fecal samples were established. Written informed consent was obtained from all participants. The inclusion and exclusion criteria for the healthy volunteers and PCOS patients were the same as those described previously [6]. Patients with PCOS were diagnosed according to the 2003 Rotterdam criteria, which require the presence of at least two of the following: oligo-ovulation and/or anovulation; clinical and/or biochemical signs of hyperandrogenism; and ultrasound findings of polycystic ovaries in 1 or 2 ovaries, ≥ 12 follicles measuring 2 to 9 mm in diameter, and/or ovarian volume ≥ 10 mL. Diagnoses of PCOS were made after the exclusion of other etiologies for hyperandrogenemium or ovulatory dysfunction including Cushing syndrome, 21-hydroxylase deficiency, thyroid disease, androgen-secreting tumors, congenital adrenal hyperplasia and hyperprolactinemia. The healthy volunteers with regular menstrual cycles, normal ovarian morphology, and normal level of androgen were from the general community. A total of eighty-eight participants were recruited between June 2019 and November 2020. The cohort was divided into four groups: Healthy-LB (Healthy individuals, BMI < 24) (*n* = 21), PCOS-LB (PCOS patients, BMI < 24) (*n* = 22), Healthy-HB (Healthy individuals, BMI ≥ 24) (*n* = 20) and PCOS-HB (PCOS patients, BMI ≥ 24) (*n* = 25). All necessary clinical parameters and DNA methylation determination were determined as previously reported [6]. All the participants were asked to come to our department during days 2–4 of the menstrual period after an overnight fast. Peripheral blood samples were collected from all subjects for the parameters measurements and then oral glucose tolerance test and insulin releasing test were performed. Further blood samples were taken at 120 min for measurement of glucose and insulin.

### 16S rRNA gene amplicon sequencing and data processing

DNA from stool samples was extracted using HiPure Stool DNA Kits B (D3141-03B, Guangzhou Meiji Biotechnology Co., Ltd., China). 16S rRNA gene amplification [24, 25], DNA library concentration validation, multiplexing and Illumina sequencing were performed as previously described [6]. Raw sequencing data were trimmed for quality and length, and Illumina adapters were removed using Fastp (version 0.19.6) with the following criteria: (i) reads containing ≥ 10% N bases; (ii) more than 50% of the base with a quality score < 20; (iii) the adaptor sequence and its subsequent sequence; (iv) truncated reads < 200 bp. If one read in each paired-end reads reaches the filtering standard, the paired reads will be removed then.The QIIME2 pipeline [26] was applied to process and analyze 16S rRNA gene sequencing data (QIIME2, version 2019.4). The sequence file processed by Fastp was imported into QIIME2 for subsequent data filtering. The filtering steps were as follows: (i) remove primers with default parameters by cutadapt function in QIIME2; (ii) DADA2 was used to remove interference sequence, chimeric sequence, etc. The parameters –p-trunc-len-f and –p-trunc-len-r were set to 0, and default parameters were used for other analysis parameters. The representative sequence and abundance table were obtained by DADA2 [27]. Taxonomy was assigned to sequences using q2-feature-classifier classifysklearn [28] against Silva [29]. Microbial community functional composition was predicted based on 16S rRNA sequences using PICRUSt software [30].

### ITS2 gene amplicon sequencing and data processing

DNA was extracted from stool samples using E.Z.N.A.® Stool DNA Kit (Omega Biotek, Norcross, GA, U.S.). The ITS2 gene was amplified by PCR with barcoded forward primers (ITS1F: 5’-CTTGGTCATTTAGAGGAAGTAA-3’) and reverse primers (ITS2R: 5’-GCTGCGTTCTTCATCGATGC-3’) [31]. Pooled DNA products were used to construct an Illumina Pair-End library following Illumina’s genomic DNA library preparation procedure, and the amplicon library was paired-end sequenced (2 × 250 bp) using the Illumina platform (Shanghai BIOZERON Biotech. Co., Ltd). The bioinformatics analysis procedure for ITS2 data was similar to that for 16S rRNA data, the UNITE (ITS) reference database was used [32].

### Serum wide targeted metabolomics profiling and data preprocessing

The serum sample extracts were analyzed using an LC–ESI–MS/MS system (UPLC, ExionLC AD, https://sciex.com.cn/; MS, QTRAP® System, https://sciex.com/). LIT and triple quadrupole (QQQ) scans were acquired with a triple quadrupole-linear ion trap mass spectrometer (QTRAP), QTRAP® LC–MS/MS System, equipped with an ESI Turbo Ion-Spray interface, operating in positive and negative ion mode and controlled by Analyst 1.6.3 software (Sciex). The ESI source operation parameters were as follows: source temperature 500 °C; ion spray voltage (IS) 5500 V (positive), -4500 V (negative); ion source gas I (GSI), gas II (GSII), curtain gas (CUR) set at 55, 60, and 25.0 psi, respectively; and high collision gas (CAD). Instrument tuning and mass calibration were performed with 10 and 100 μmol/L polypropylene glycol solutions in QQQ and LIT modes, respectively. A specific set of MRM transitions was monitored for each period according to the metabolites eluted within this period.

### Statistical analysis

All statistical tests were performed using R (version 4.0.2). The α diversity index was calculated using the R program package ‘vegan’ (version 2.5.7). Group comparisons were conducted by ANOVA tests, and the LDuncan method (package laercio, 1.0–1) was used to group differences. Differences in community were determined by principal co-coordinates analysis (PCoA) and RDA (package vegan, 2.5.7). PERMANOVA and ANOSIM were conducted to assess statistical significance. Mantel tests were carried out to examine Spearman’s rank correlation between bacterial, fungal, and functional pathways, metabolites and the clinical index matrix (vegan package). Linear discriminant analysis effect size (LEfSe) analysis was performed [33, 34]. Kruskal–Wallis tests among multiple groups and Wilcoxon tests between paired groups were conducted. Multiple hypothesis tests were adjusted using the Benjamini-Hochberg (B-H) false discovery rate (FDR).

Metabolome data were analyzed by PLS-DA with the package mixOmics (6.14.1). Characterized metabolites were screened out based on (1) variable importance in projection (VIP) > 1, (2) fold change of > 2 or < 0.5 and (3) FDR adjusted *p* < 0.05 (Wilcox test). Randomforest [35] algorithms were trained with the multiomics data using the randomForest R package [36] and graphed by pROC package. Input features were excavated on the basis of Wilcox test comparison and the mean decrease in Gini by random forest importance parameter evaluation. Data were assigned to training (80%) and test (20%) datasets after the whole dataset was shuffled. Subsequently, we further explored the complicated network interaction of discriminative features derived from multiomics data using Cytoscape (v3.5.1).

## Results

### Clinical characteristics of the participants

**Fig. 1:**
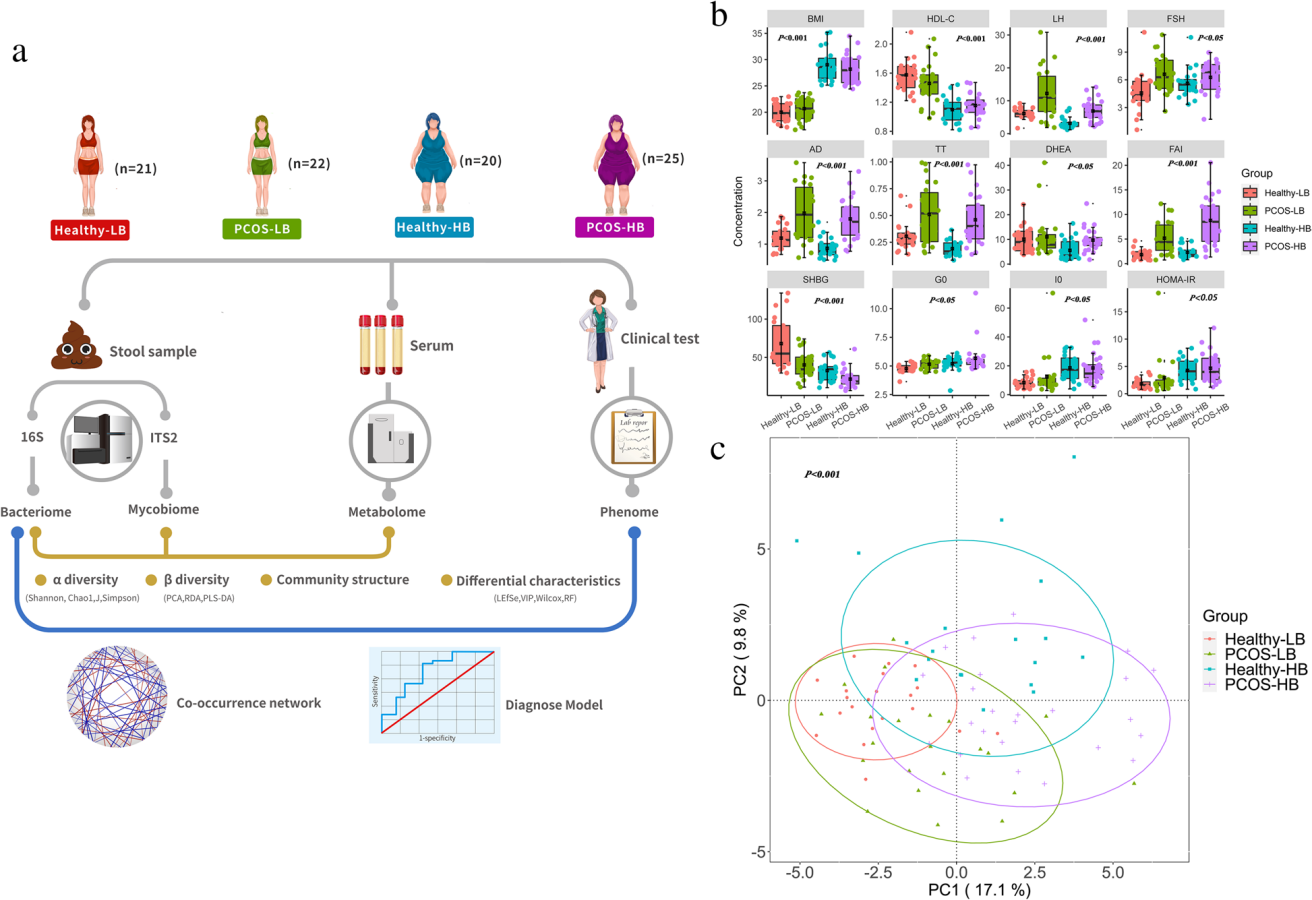
Clinical parameter distribution. **a** Overview of the study design. **b** Differences in clinical index. Data are shown as the mean ± SD, and an error bar is shown. *p* values denote the significance among groups. Letters indicate ANOVA grouping. **c** Differences in clinical index structures as revealed by principal co-ordinates analysis (PCoA)

The participant design and demographics are shown in Fig. 1a and Table 1. Compared with the Healthy group, PCOS patients displayed significantly higher luteinizing hormone (LH), follicle-stimulating hormone (FSH), estrogen (E2), androstenedione (AD), total testosterone (TT), free androgen index (FAI), glucose level at fasting status (G0), insulin level at 120 min after glucose load (I120), and glucose level at 120 min after glucose load (G120) while lower progesterone (PROG) and sex hormone-binding globulin (SHBG) (FDR adjusted *p* < 0.05) (Table S1). PCOS-HB patients (PCOS patients, BMI ≥ 24) had significantly higher LH, LH/FSH, AD, TT, dehydroepiandrosterone (DHEA), and FAI while lower SHBG than that of Healthy-HB subjects (FDR adjusted *p* < 0.05) (Fig. 1b, Table 1). There were significant differences in the clinical parameter structure among the four groups based on PCoA (Fig. 1c) (PERMANOVA: *p* < 0.001; ANOSIM: *p* < 0.05).

**Table 1:** Clinical characteristics baseline in the BMI-related healthy and PCOS groups

Parameter	Characteristic	Healthy-LB (*n* = 21)	PCOS-LB (*n* = 22)	*p* value	FDR	Healthy-HB (*n* = 20)	PCOS-HB (*n* = 25)	*p* value	FDR	Four groups *p* value	Four groups FDR
Demographic characteristics	Age (years)	28.53 ± 3.19	28.96 ± 3.87	0.6	0.832	29.9 ± 2.98	29.04 ± 3.89	0.283	0.498	0.555	0.666
	BMI (kg/m^2^)	20.01 ± 1.83	20.68 ± 2.17	0.23	0.504	29.03 ± 3.06	28.19 ± 2.82	0.332	0.541	**0.001**	**0.001**
Liver function	LDH (U/L)	174.2 ± 29.51	174.96 ± 44.35	0.942	0.972	183.8 ± 30.79	198.88 ± 30.71	0.121	0.272	**0.013**	**0.025**
	AST (U/L)	20.2 ± 6.09	20.1 ± 5.73	0.808	0.972	19.62 ± 4.9	24.88 ± 10.51	0.071	0.209	0.121	0.166
	ALT (U/L)	14.77 ± 7.95	18.19 ± 11.92	0.526	0.827	19.96 ± 10.31	32.35 ± 26.24	**0.043**	0.144	**0.002**	**0.004**
	GGT (U/L)	20 ± 9.69	20.91 ± 9.58	0.551	0.832	21.97 ± 9.72	28.92 ± 14.32	**0.044**	0.144	**0.021**	**0.039**
	ALP (U/L)	69.67 ± 16.8	69 ± 16.12	0.444	0.748	76.29 ± 16.11	78.04 ± 23.73	0.592	0.723	0.114	0.162
	CHE (U/mL)	7.32 ± 1.49	7.96 ± 1.35	0.294	0.579	9.02 ± 1.32	9.6 ± 1.54	0.371	0.583	**0.001**	**0.001**
	MAO (U/L)	3.29 ± 0.91	3 ± 1.12	0.303	0.579	5.42 ± 2.74	4.1 ± 1.47	0.128	0.272	**0.001**	**0.001**
	AFU (U/L)	23.34 ± 4.34	27.23 ± 6.57	**0.043**	0.142	26.95 ± 5.69	29.32 ± 6.5	0.326	0.541	**0.031**	0.056
	TP (g/L)	76.28 ± 4.51	77.01 ± 3.86	0.972	0.972	76.77 ± 4.77	76.56 ± 3.83	0.955	1	0.998	0.998
	ALB (g/L)	44.1 ± 2.12	44.79 ± 1.94	0.338	0.619	45.69 ± 6.92	44.74 ± 2.14	0.53	0.707	0.676	0.782
	GLB (g/L)	32.19 ± 3.03	32.22 ± 2.89	0.674	0.899	32.41 ± 3.92	31.82 ± 2.79	0.576	0.723	0.952	0.987
	ALB/GLB	1.38 ± 0.11	1.4 ± 0.12	0.601	0.832	1.39 ± 0.18	1.41 ± 0.15	0.479	0.68	0.822	0.928
	TBIL (μmol/L)	10.87 ± 4.56	10.94 ± 4.37	0.971	0.972	11.84 ± 5.65	10.45 ± 2.93	0.743	0.861	0.932	0.987
	DBIL (μmol/L)	2.05 ± 0.94	1.96 ± 0.81	0.933	0.972	2.51 ± 2.4	1.77 ± 0.57	0.15	0.3	0.56	0.666
	IBIL (μmol/L)	8.82 ± 3.7	8.99 ± 3.67	0.846	0.972	9.33 ± 3.75	8.67 ± 2.46	1	1	0.964	0.987
Renal function	CO_2_ (mmol/L)	25.17 ± 2.48	26.22 ± 2.19	0.178	0.421	25.42 ± 7.61	25.62 ± 2.56	0.098	0.239	0.099	0.15
	BUN (mmol/L)	4.8 ± 1.28	4.09 ± 0.71	**0.032**	0.12	4.74 ± 0.94	4.59 ± 1.14	0.418	0.634	0.067	0.114
	Cr (μmol/L)	75.62 ± 7.22	71.19 ± 8.67	0.103	0.282	72.5 ± 11.08	71.68 ± 9.5	0.561	0.723	0.105	0.153
Metabolic index	UA (μmol/L)	346.39 ± 89.7	355.47 ± 74.5	0.605	0.832	404.96 ± 138.48	428.22 ± 79.55	0.234	0.428	**0.005**	**0.009**
	TC (mmol/L)	4.74 ± 0.83	4.74 ± 0.61	0.913	0.972	4.84 ± 0.72	4.8 ± 0.87	0.508	0.699	0.884	0.972
	TG (mmol/L)	0.97 ± 0.39	1.06 ± 0.45	0.452	0.748	1.37 ± 0.65	1.43 ± 0.78	0.991	1	**0.049**	0.085
	HDL-C (mmol/L)	1.58 ± 0.22	1.46 ± 0.29	0.096	0.282	1.1 ± 0.18	1.16 ± 0.16	0.226	0.428	**0.001**	**0.001**
	LDL-C (mmol/L)	2.89 ± 0.66	2.94 ± 0.42	0.762	0.972	3.18 ± 0.58	3.15 ± 0.67	0.811	0.91	0.24	0.301
Sex hormone	LH (mIU/mL)	6.12 ± 1.65	12.23 ± 7.73	**0.004**	**0.027**	3.17 ± 1.69	6.89 ± 3.29	**0.001**	**0.001**	**0.001**	**0.001**
	FSH (mIU/mL)	4.58 ± 2.11	6.6 ± 2.04	**0.001**	**0.015**	5.64 ± 1.59	6.27 ± 1.68	0.085	0.225	**0.002**	**0.003**
	LH/FSH	2.05 ± 3.15	1.93 ± 1.16	0.156	0.402	0.56 ± 0.2	1.23 ± 0.95	**0.001**	**0.002**	**0.001**	**0.001**
	PRL (ng/mL)	26.15 ± 16.61	15.84 ± 7.79	**0.015**	0.064	20.5 ± 13.46	18.15 ± 10.28	0.624	0.742	0.092	0.146
	E2 (pg/mL)	28.58 ± 18.32	69.08 ± 50.55	**0.001**	**0.001**	32.97 ± 14.52	44.13 ± 15.74	**0.033**	0.144	**0.001**	**0.001**
	PROG (nmol/L)	0.9 ± 0.3	0.64 ± 0.37	**0.008**	**0.039**	0.64 ± 0.44	0.54 ± 0.48	0.087	0.225	**0.001**	**0.003**
Androgen	AD (ng/mL)	1.2 ± 0.37	1.99 ± 0.9	**0.004**	**0.027**	0.87 ± 0.32	1.8 ± 0.65	**0.001**	**0.001**	**0.001**	**0.001**
	TT (ng/mL)	0.31 ± 0.13	0.51 ± 0.28	**0.033**	0.12	0.19 ± 0.08	0.46 ± 0.23	**0.001**	**0.001**	**0.001**	**0.001**
	DHEA (ng/mL)	10.11 ± 5.27	11.02 ± 8.88	0.953	0.972	5.57 ± 4.41	9.72 ± 4.71	**0.005**	**0.031**	**0.004**	**0.008**
	FAI	1.83 ± 0.99	5.18 ± 3.35	**0.001**	**0.002**	2.32 ± 1.27	8.78 ± 4.93	**0.001**	**0.001**	**0.001**	**0.001**
	SHBG (nmol/L)	68.1 ± 31.72	40.3 ± 17.5	**0.002**	**0.022**	32.91 ± 13.34	21.75 ± 11.81	**0.005**	**0.031**	**0.001**	**0.001**
Glucose tolerance	G0 (mmol/L)	4.77 ± 0.38	5.16 ± 0.42	**0.005**	**0.027**	5.19 ± 0.69	5.67 ± 1.34	0.479	0.68	**0.001**	**0.001**
	G120 (mmol/L)	5.76 ± 1.33	7.41 ± 2.44	**0.007**	**0.039**	6.98 ± 1.68	9.18 ± 4.09	**0.023**	0.123	**0.001**	**0.001**
Insulin	I0 (mIU/L)	8.33 ± 3.65	12.36 ± 14.26	0.459	0.748	18.63 ± 9.21	18.52 ± 10.68	0.848	0.91	**0.001**	**0.001**
	I120 (mIU/L)	52.3 ± 34.95	92.41 ± 78.14	**0.046**	0.142	106.17 ± 85.51	134.32 ± 71.02	0.13	0.272	**0.001**	**0.002**
	HOMA-IR	1.8 ± 0.87	2.94 ± 3.73	0.269	0.564	4.27 ± 2.13	4.67 ± 2.7	0.847	0.91	**0.001**	**0.001**
Methylation	FKBP5-Met1	51.86 ± 3.4	50.61 ± 2.41	0.182	0.421	54.59 ± 6.13	51.24 ± 4.78	**0.041**	0.144	0.093	0.146
	FKBP5-Met2	80.41 ± 4.41	80.51 ± 4.07	0.857	0.972	84.81 ± 8.54	79.72 ± 7.3	**0.046**	0.144	0.161	0.208
	FKBP5-Met	66.14 ± 3.64	65.56 ± 2.67	0.839	0.972	69.7 ± 7.06	65.48 ± 5.79	**0.045**	0.144	0.153	0.203

The data are shown as the mean ± SD

*BMI* Body mass index, *LDH* Lactate dehydrogenase, *AST* Aspartate aminotransferase, *ALT* Alanine aminotransferase, *GGT* Glutamyltransferase, *ALP* Alkaline phosphatase, *CHE* Cholinesterase, *MAO* Monoamine oxidase, *AFU* α-L-fucosidase, *TP* Total protein, *ALB* Albumin, *GLB* Globulin, *TBIL* Total bilirubin, *DBIL* Direct bilirubin, *IBIL* Indirect bilirubin, *BUN* urea nitrogen, *Cr* Creatinine, *UA* Uric acid, *TG* Triglycerides, *TC* Total cholesterol, *HDL-C* High-density lipoprotein cholesterol, *LDL-C* Low-density lipoprotein cholesterol, *LH* Luteinizing hormone, *FSH* Follicle stimulating hormone, *PRL* Prolactin, *E2* Estrogen, *PROG* Progesterone, *AD* Androstenedione, *TT* Total testosterone, *DHEA* Dehydroepiandrosterone, *FAI* free androgen index, *SHBG* Sex hormone-binding globulin, G0 glucose level at fasting status, *G120* Glucose level at 120 min after glucose load, I0 insulin level at fasting status, I120 insulin level at 120 min after glucose load, *HOMA-IR* homeostasis model assessment for IR, *FKBP5-Met1* FKBP5 DNA methylation at *CpG* 35,657,180/hg19, *FKBP5-Met2 FKBP5 DNA* methylation at CpG 35,657,202/hg19, *FKBP5*-Met average of FKBP5 DNA methylation at CpG 35,657,180 and 35,657,202/hg19

### Altered bacterial diversity and community in PCOS patients

**Fig. 2:**
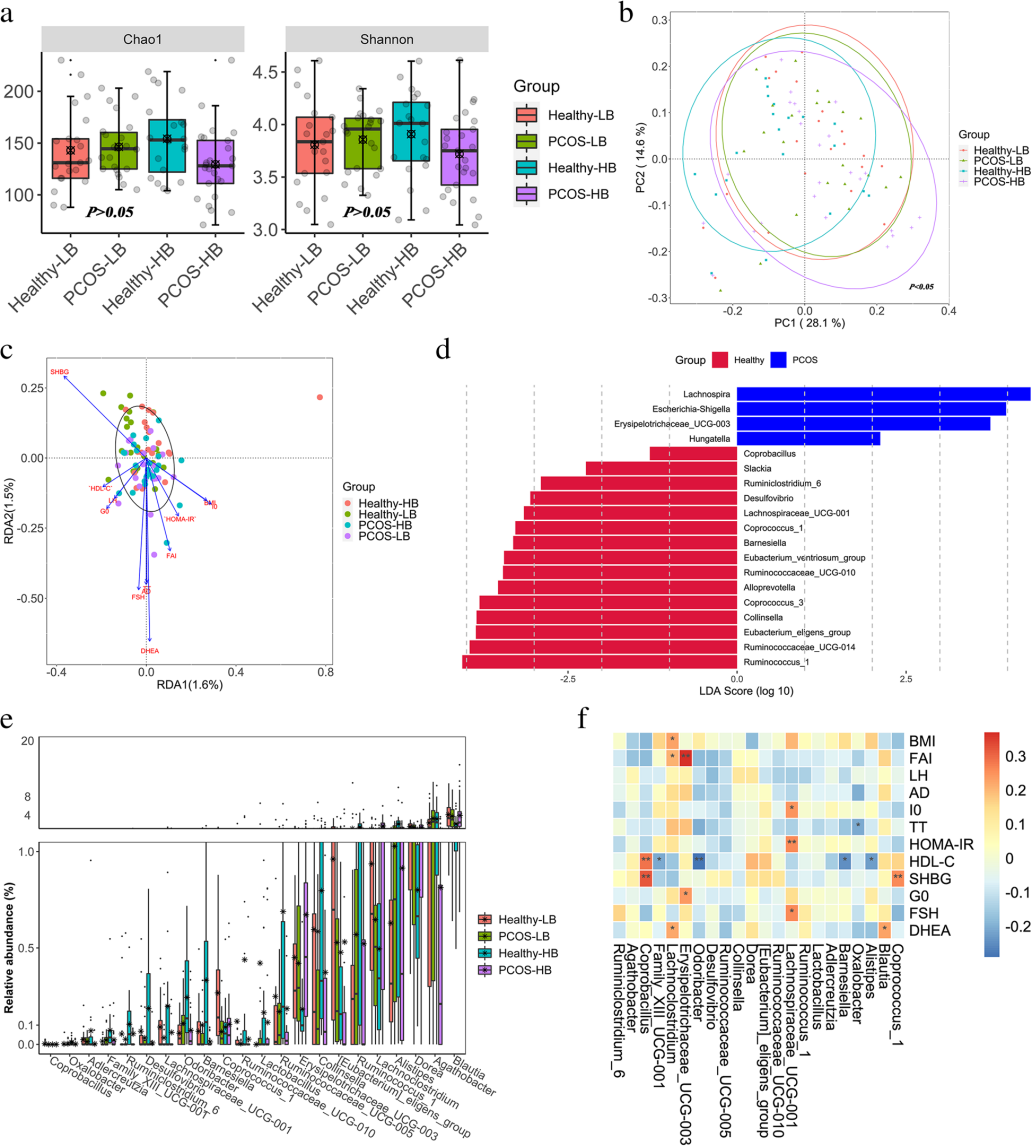
Fecal bacterial characteristic variations associated with PCOS. **a** α diversity. *p* values denote the significance among groups. Letters indicate ANOVA grouping. **b** Differences in bacterial structures as revealed by PCoA analysis. **c** RDA analyses reflecting differences in gut microbiota structures fitted with significantly correlated clinical properties. **d** Characteristic bacterial taxa based on LDA effect size (LEfSe) analysis between PCOS patients and healthy individuals. **e** The distinguished bacterial genera screened by Kruskal–Wallis tests. **f** The heatmap depicts the relationship between distinguished bacterial genera (screened by Kruskal–Wallis tests) and key clinical parameters

An average of 143 observed OTUs/sample were obtained from 88 samples after 16S rRNA gene sequencing (Table S2). Significantly reduced α-diversity was observed in PCOS-HB compared with Healthy-HB (*p* < 0.05) (Fig. 2a). There were no significant relationships between the bacterial diversity index and key clinical variables(*p* > 0.05) (Table S3). PCoA revealed significant differences (Fig. 2b) among the groups (PERMANOVA: *p* < 0.05; ANOSIM, *p* < 0.05). There were no significant correlations between key clinical variables and bacterial community (*p* > 0.05) (Fig. 2c).Firmicutes, Bacteroidetes, Proteobacteria and Actinobacteria accounted for more than 90% of the total phylum (Figure S1a). *Bacteroides*, *Prevotella*_9, *Faecalibacterium*, and *Roseburia* were the dominant bacterial genera (Figure S1b). LEfSe analysis revealed *Ruminococcus torques*,*Escherichia*/*Shigella*, *Allisonella*, *Eggerthella* and *Hungatella* as PCOS-featured genera (Fig. 2d). *Lactobacillus, Coprococcus_*1*, Coprococcus_*3 and *Catenibacterium* showed suggestive differences between Healthy-LB and PCOS-LB (*p* < 0.05) (Table S4, Figure S2a). Moreover, genus comparison between Healthy-HB and PCOS-HB by the Wilcoxon signed-rank test and LEfSe analysis revealed 25 significant taxa (Table S4, Figure S2b). Bacterial taxa enriched in Healthy-LB were *Blautia* (4.10%), *Dorea* (1.50%), whereas *Agathobacter* (3.28%) and *Lactobacillus* (0.42%) were enriched in PCOS-LB patients. In the Healthy-HB group, *Alistipes* (2.10%), *Ruminococcus*_1 (1.38%) were enriched. *Lachnoclostridium* (1.66%) and Erysipelotrichaceae_UCG-003 (0.67%) were overrepresented in the PCOS-HB group (Fig. 2e). *Coprobacillus* and *Coprococcus_*1 were significantly positively related to SHBG (*p* < 0.05), and HOMA-IR displayed a positive correlation with Lachnospiraceae_UCG-001 (Fig. 2f).

### Altered fecal fungal profile in PCOS patients

**Fig. 3:**
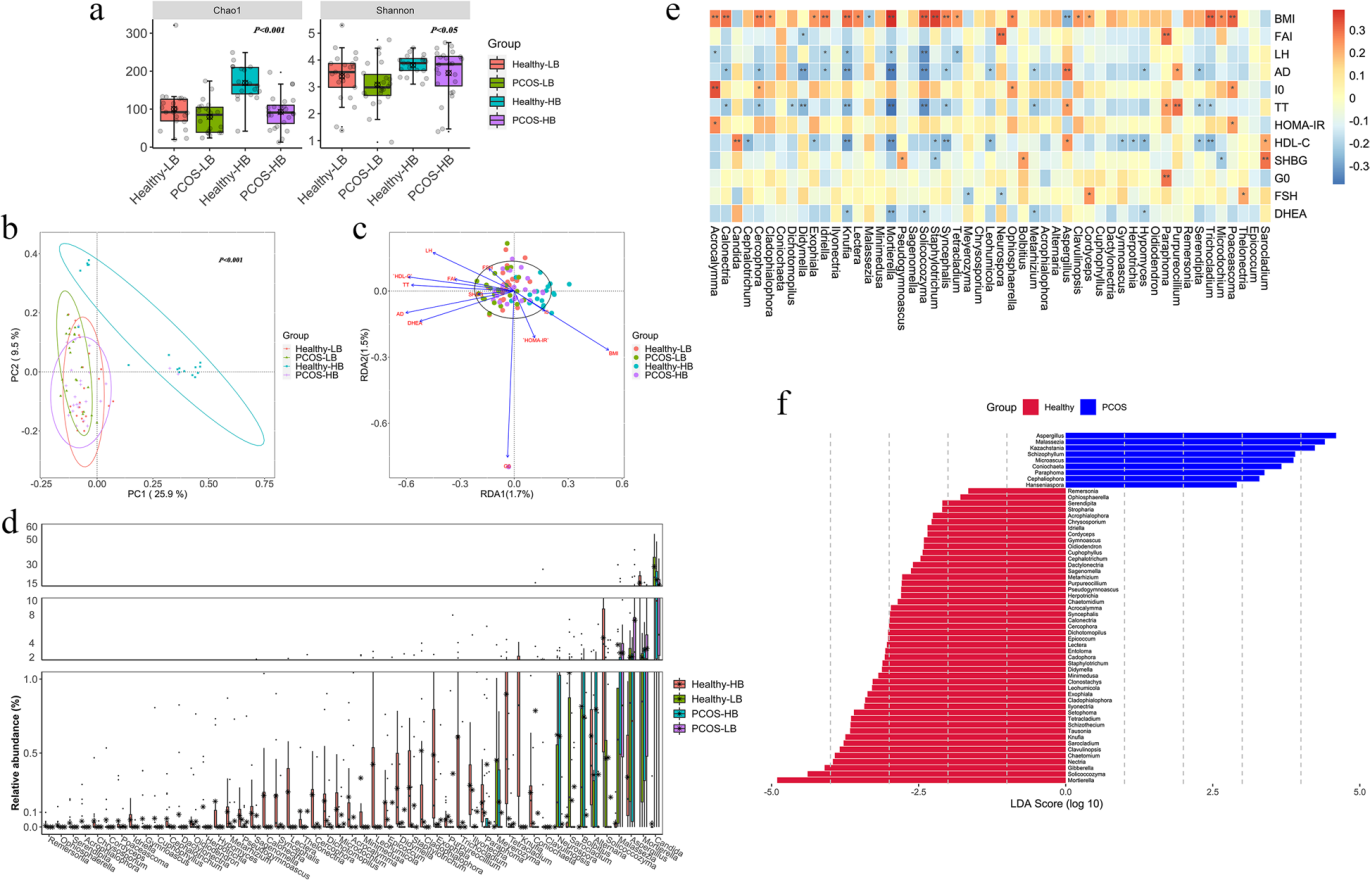
Fecal fungal characteristic variations associated with PCOS. **a** α diversity measured by Chao1 and Shannon indices was highest in Healthy-HB. *p* values denote the significance among groups. Letters indicate ANOVA grouping. **b** Differences in fungal structures among Healthy-LB, Healthy-HB, PCOS-HB and PCOS-LB, as revealed by PCoA analysis. **c** RDA analyses reflecting differences in gut fungal structures fitted with significantly correlated clinical properties. **d** Distinguished fungal genera screened by Kruskal–Wallis tests. **e** Correlation between distinguished fungal genera and key clinical parameters. **f** Characteristic fungal taxa based on LDA effect size (LEfSe) analysis between PCOS patients and healthy individuals

Healthy-HB exhibited significantly higher Chao1 and Shannon indices than PCOS-LB (Fig. 3a). A significantly positive relationship for BMI and negative relationships for FAI, LH, AD, TT, HDL-C and DHEA with observed OTUs and Chao1 were detected (*p* < 0.05) (Table S3). Overall, there were significant alterations in the gut fungal community among the four groups (Fig. 3b) (PERMANOVA: *p* < 0.05; ANOSIM: *p* < 0.05). TT exerted a significantly higher influence on the fungal community structure (*r*^2^ = 0.08, *p* < 0.05) (Fig. 3c). Ascomycota, Basidiomycota, and Mortierellomycota were the dominant fungal phyla and Mortierellomycota was uniquely higher in Healthy-HB (Figure S3a). Fungal genera, *Candida* depletion while *Fusarium*, *Mortierella* and *Solicoccozyma* enrichment was observed in Healthy-HB (Figure S3b). Multiple group comparison revealed 16 significantly differential fungal genera in Healthy-LB versus PCOS-LB and 75 taxa in Healthy-HB versus PCOS-HB (Fig. 3d, Table S5). *Candida* was positively related to HDL-C, and *Mortierella* was negatively related to BMI but adversely to LH, AD, TT, HDL-C and DHEA (Fig. 3e). PCOS featured fungal indicators in genus level were *Candida*, *Malassezia*, *Kazachstania*, *Microascus*, *Coniochaeta*, *Xepicula*, *Paraphoma*, *Pyrenochaetopsis*, *Cephaliophora*, *Epicoccum* and *Sclerophora* (Fig. 3f). PCOS-HB had more distinguished fungal genera as indicators than that of PCOS-LB (Figure S4,S5).

### Serum metabolomics alterations in PCOS patients

**Fig. 4:**
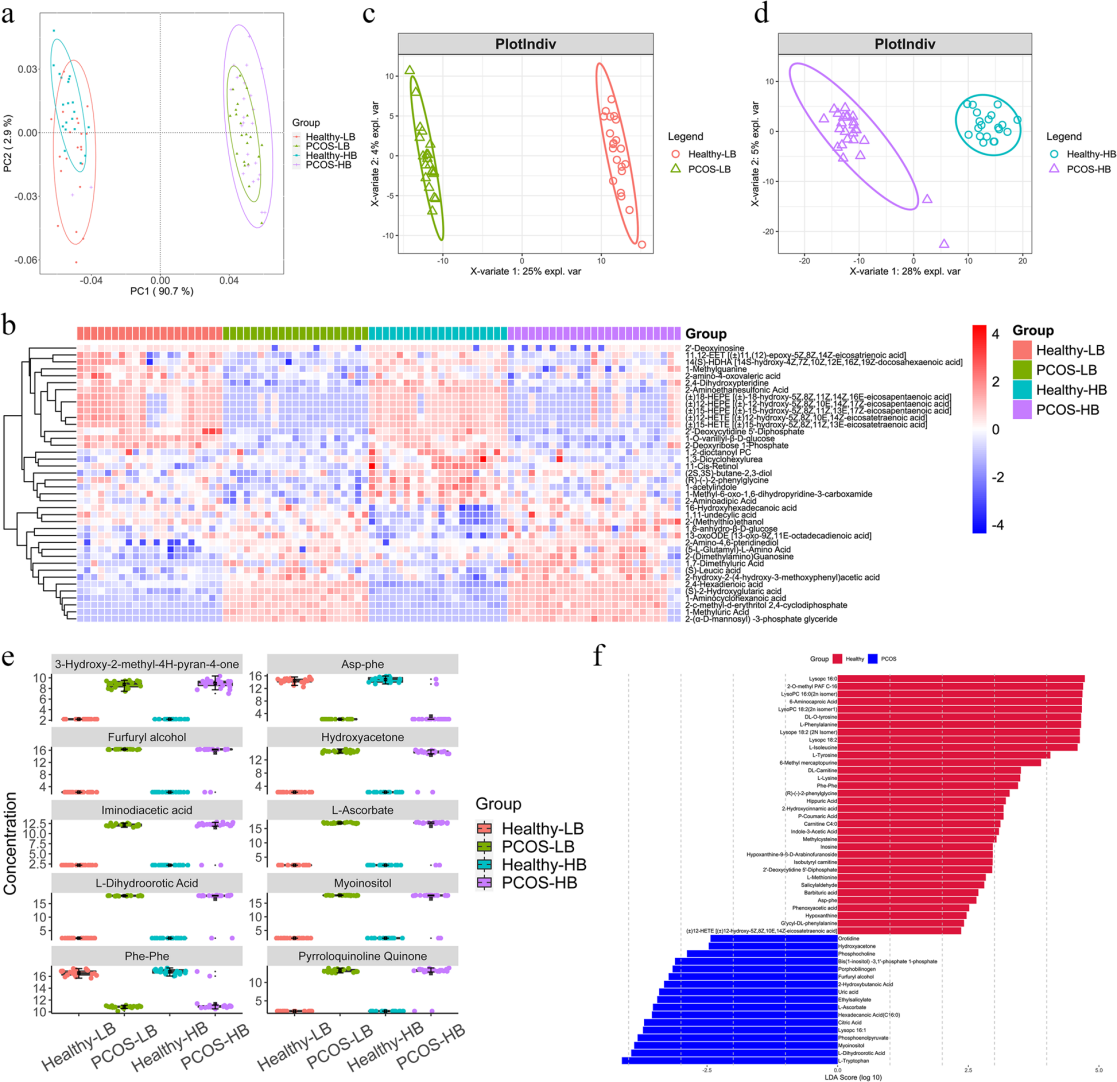
Serum metabolite variations associated with PCOS. **a** Differences in fungal structures as revealed by PCoA analysis. **b** Heatmap showing the relative abundance of the 40 metabolites screened by Kruskal–Wallis tests. **c** PLS-DA plot revealing the differential metabolite pattern between Healthy-LB and PCOS-LB. **d** PLS-DA plot revealing the differential metabolite pattern between Healthy-HB and PCOS-HB. **e** The distribution of the top 10 metabolites as VIP values ranked as shared by comparison between Healthy-LB and PCOS-LB and between Healthy-HB and PCOS-HB. **f** Characteristic metabolites based on LDA effect size (LEfSe) analysis between PCOS patients and healthy controls

A total of 601 metabolites were identified and quantified, with significant differences among the four groups (PERMANOVA:*p* < 0.05; ANOSIM: *p* < 0.05) (Fig. 4a). BMI, FAI, LH, AD, TT, SHBG and DHEA exerted significantly higher influences on the metabolite community structure (*p* < 0.05) (Figure S6a). Multiple group comparisons revealed 284 significantly differential metabolites in Healthy-LB versus PCOS-LB and 358 metabolites in Healthy-HB versus PCOS-HB (*p* < 0.05) (Table S6). Metabolite indicators associated with PCOS, PCOS-LB and PCOS-HB were also revealed by LEfSe analysis (Fig. 4f, Figure S7, S8). The healthy and PCOS groups showed totally different metabolite patterns (Fig. 4b, Figure S6 b,c,d). VIP scores obtained by PLS-DA analysis (partial least squares discriminant analysis) (Fig. 4c, 4d), together with the threshold of FDR adjusted *p* < 0.05 and |log_2_FC|> 1, were set as thresholds to screen out featured metabolites. Ten commonly featured serum metabolites of the top VIP value were both observed between comparison pairs of Healthy-LB versus PCOS-LB and Healthy-HB versus PCOS-HB. 3-Hydroxy-2-methyl-4H-pyran-4-one, furfuryl alcohol, iminodiacetic acid, L-dihydroorotic acid, hydroxyacetone, L-ascorbate, myoinositol and pyrroloquinoline quinone were enriched in PCOS patients, and Phe-Phe and Asp-Phe were abundant in healthy individuals (Fig. 4e).

### Integrative multiomic signatures in the diagnosis of PCOS

**Fig. 5:**
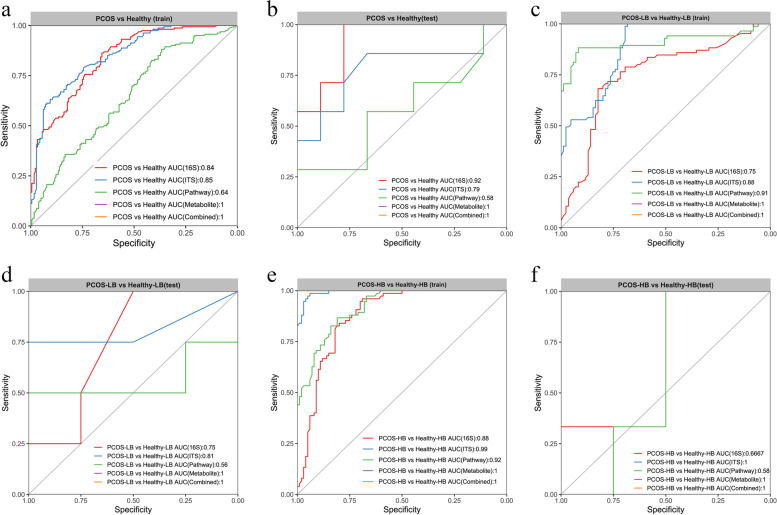
Disease status classification using disease-associated taxa and/or metabolites. (**a**, **c**, **e**) Random forest classifiers were constructed to discriminate PCOS and healthy, PCOS-LB and Healthy-LB, PCOS-HB and Healthy-HB, respectively, in the training dataset. (**b**, **d**, **f**) Random forest classifiers composed of bacterial and fungal genera, metabolites, predicted pathways and their combinations were constructed to discriminate PCOS and healthy, PCOS-LB and Healthy-LB, PCOS-HB and Healthy-HB in the test dataset. ROC, receiver operating characteristic curve. AUC, area under the curve. The input features were excavated on the basis of Wilcox test comparison and the mean decrease in Gini by random forest importance parameters. Data were assigned to training (80%) and test (20%) datasets after the whole dataset was shuffled

We built random forest models based on multiomics characteristics individually or their combination to discriminate PCOS patients, PCOS-LB patients and PCOS-HB patients from the corresponding healthy individuals. A five-fold cross-validated random forest model was further employed to select key discriminatory characteristics after first screening by Wilcoxon signed-rank test comparison (Figure S9, Figure S10). This method identified 9 bacterial genera, 4 predicted pathways, 11 fungal genera and the top 30 metabolites that distinguished PCOS from healthy individuals with AUCs of 0.84, 0.64, 0.85 and 1, respectively, and their combination also reached an AUC of 1 in the training dataset (Fig. 5a). Robust efficacy was verified by fungal taxa and metabolites in the test dataset (Fig. 5b). Similarly, the metabolite-derived model was more accurate than the microbe-based model in discriminating PCOS-LB from Healthy-LB (metabolites, AUC = 1; 16S, AUC = 0.75; predicted pathway, AUC = 0.91; ITS2, AUC = 0.88) (Fig. 5c). Functional pathway performed weaker in the test dataset (Fig. 5d). The use of 16S and predicted pathway data showed good performance in distinguishing PCOS-HB from Healthy-HB (AUC = 0.88 and 0.92, respectively), but poor performance (AUC = 0.67 and 0.58) was observed in the test dataset (Fig. 5e, 5f). The findings indicated that metabolites alone can achieve great performance in distinguishing disease from health, much better than microbiota-driven features. Nevertheless, fungal features performed better than bacterial genera in discriminating PCOS from health.

### Multiomic data interaction associated with PCOS patients

The Mantel test indicated that only the metabolite distance matrix, and not 16S or ITS2, predicted pathway distance, remained significantly positively correlated with phenotypic distance (*r* = 0.15, *p* = 0.001) (Table S7). Although there was a significantly positive relationship between the 16S and ITS2 data matrices (*r* = 0.07, *p* = 0.015), the bacterial diversity showed no significant linear correlation with fungal diversity (Figure S11).

**Fig. 6:**
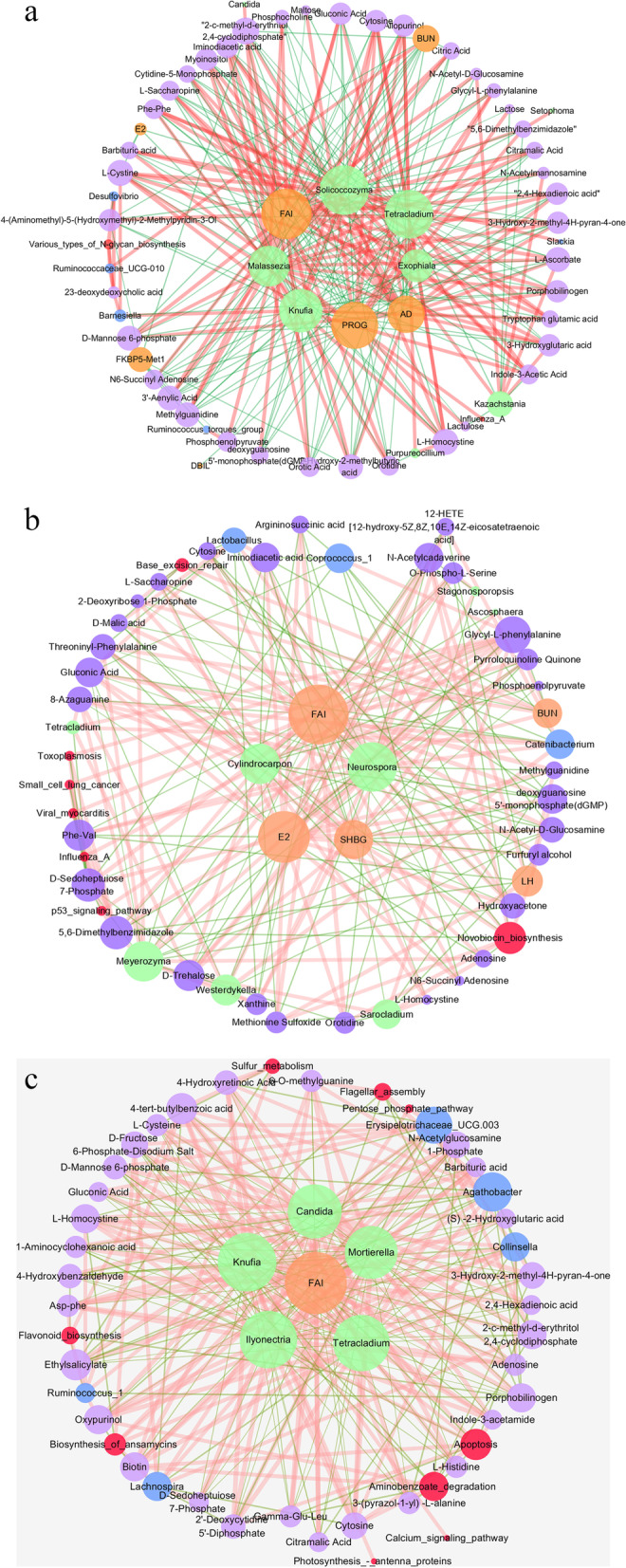
Integrative co-occurrence network reflecting multiomic-phenotype interactions. (**a,b,c**) Network revealed both significant (*p* < 0.05) and suggestive correlations (∣r∣ > 0.4, Spearman analysis) between differentially abundant bacterial, fungal, predicted pathway, metabolites and clinical indices in PCOS and healthy, PCOS-LB and Healthy-LB, PCOS-HB and Healthy-HB. Nodes represent characteristics. Purple, blue, red, green and yellow nodes denote metabolites, bacterial taxa, predicted pathways, fungal taxa and clinical parameters. Lines connecting nodes indicate positive (red) or negative (green) correlations

We tested for the correlations of different multiomics characteristics derived from the above-mentioned diagnostic study based on Spearman’s correlation test (∣*r*∣ > 0.4). In healthy vs. PCOS, fecal bacteria, fungi, and predicted pathways displayed more associations with the clinical indices FAI, PROG and AD. For example, higher levels of FAI were accompanied by increased cytidine-5-monophosphate (*p* < 0.05, *r* = 0.67) and iminodiacetic acid (*p* < 0.05, *r*= 0.63) and lower allopurinol (*p* < 0.05, *r* = -0.63), lactulose (*p* < 0.05,  *r*= -0.62), lactose (*p* < 0.05, *r* = -0.62) and maltose (*p* < 0.05, *r* = -0.62) levels. The fungal taxa *Solicoccozyma*, *Tetracladium*, *Malassezia*, *Knufia* and *Exophiala* were also center-connected (Fig. 6a). Therefore, higher levels of *Solicoccozyma* were negatively related to 2,4-hexadienoic acid but positively to L-homocystine. *Malassezia* showed negative correlations with L-homocystine (*p* < 0.05, *r* = -0.59) but positive correlations with 2-c-methyl-d-erythritol 2,4-cyclodiphosphate (*p* < 0.05, *r* = 0.54).

In Healthy-LB vs. PCOS-LB, we observed that many metabolic features were associated with FAI, E2 and SHBG. For example, FAI was positively associated with methylguanidine (*p* < 0.05, *r* = 0.76) and hydroxyacetone (*p* < 0.05, *r* = 0.70) but negatively correlated with 2-deoxyribose 1-phosphate (*p* < 0.05, *r* = -0.70). Featured bacterial taxa, *Catenibacterium*, *Coprococcus*_1 and *Lactobacillus*, were highly related to *Westerdykella*, gluconic acid and xanthine individually. The fungal taxa *Cylindrocarpon* and *Neurospora* exhibited positive and negative relationships with D-malic acid (Fig. 6b). With regard to Healthy-HB vs. PCOS-HB, increased *Candida* was linked to higher levels of 3-hydroxy-2-methyl-4H-pyran-4-one (*p* < 0.05, *r* = 0.87) and 2-c-methyl-d-erythritol 2,4-cyclodiphosphate (*p* < 0.05, *r* = 0.83) and lower levels of barbituric acid (*p* < 0.05, *r* = -0.82). *Mortierella* showed a highly negative correlation with D-fructose 6-phosphate-disodium salt (*p* < 0.05, *r* = -0.82) and D-mannose 6-phosphate (*p* < 0.05, *r* = -0.82) but a positive correlation with cytosine (*p* < 0.05, *r* = 0.83) and barbituric acid (*p* < 0.05, *r* = 0.75). The FAI was negatively related to *Agathobacter* (*p* < 0.05, *r* = -0.53) but positively with Erysipelotrichaceae_UCG-003 (*p* < 0.05, *r* = 0.43) (Fig. 6c).

## Discussion

In this study, we outlined landscapes and interaction networks of clinical indices, differential gut bacteria, fungi and serum metabolites in PCOS patients with different BMIs. According to integrated analysis of multiomics data, the most important index is FAI, which is used as a common indicator of hyperandrogenemia [37]. Overall, androgen plays an important leading role in the changes in gut bacteria, gut fungi and metabolites in patients with PCOS. Moreover, we identified and independently validated a combinatorial marker panel that was able to distinguish PCOS from non-PCOS subjects with high accuracy.

*Saccharomyces cerevisiae, Malassezia restricta, Candida albicans, Candida sake, Cyberlindnera jadinii, Cladosporium spp., Penicillium spp.,* and *Galactomyces candidum* are the most prevalent fungi in the human gut [38]. Mar Rodríguez et al. [16] showed that patients with and without obesity could be distinguished by their specific fungal composition. *Candida*, *Nakaseomyces* and *Penicillium* were the most abundant genera detected in patients with obesity, and *Mucor* was the most prevalent genera in patients without obesity. Chacón et al. [39] paid attention to the compositions of *Mucor spp*. and found that subjects with obesity and undetectable *Mucor* *spp*. showed a significantly worse cardiovascular risk profile. Research on gut fungi in metabolic diseases is still very limited to date. Jayasudha et al. [40] showed that Mucoromycota was the only phylum showing significantly decreased abundance in T2DM compared to control mycobiomes, and the median abundance of *Candida* along with *Cladosporium*, *Kodamaea*, *Meyerozyma* and *Mortierella* were increased in people with T2DM. In our study, Ascomycota*,* Basidiomycota*,* and Mortierellomycota were the dominant fungal phyla, and Mortierellomycota was uniquely higher in healthy HB individuals than in the other groups. Reduced fungal family diversity has been demonstrated in individuals with obesity prepiously [16]. In our study, Non-PCOS patients with overweight/obesity seemed less metabolically abnormal than those with PCOS, and whether the composition of different gut fungi exerts metabolic protection needs to be further assessed. Overall, the human gut mycobiome has been poorly studied and characterized in patients with PCOS. Our study showed that PCOS patients are featured in fungal indicators, such as *Candida, Malassezia, Kazachstania, Microascus, Coniochaeta, Xepicula, Paraphoma, Pyrenochaetopsis, Cephaliophora*, *Epicoccum* and *Sclerophora*, while PCOS-HB patients having more distinguished fungal genera as indicators.

Several factors in the host will have an effect on mycobiome composition and variations, including genotype, physiology, immune system, and lifestyle [41, 42]. Among them, diet is an important factor [43]. For example, *Methanobrevibacter* and *Candida* are positively associated with diets high in carbohydrates but negatively associated with diets high in amino acids, protein, and fatty acids [44]. A plant-based diet is also associated with enrichment in *Candida spp.*, whereas an animal-product-based diet is associated with enrichment in *Debaryomyces spp*. and *Penicillium spp* [43]. It is widely recognized that gut fungal diversity is significantly lower than bacterial diversity [45], but each fungal cell genome is approximately 100-fold larger than that of bacterial cells, which represents a significant biomass with numerous functions [46]. Bacteria are fundamental to maintain a balanced gut microbiota and to avoid fungal overgrowth [46], whereas imbalance in gut fungi leads to an abnormal composition of gut bacteria. Hoffmann et al. [44] reported that *Candida* and *Saccharomyces* are both positively associated with *Methanobrevibacter* and that both fungal genera are negatively associated with *Nitrososphaera.* Wheeler et al.[10] showed that fungi, including *Penicillium brevicompactum* and *Candida tropicalis*, are significantly decreased with antifungal treatment in mice but that this treatment leads to relative expansion of *Aspergillus amstelodami*, *Epicoccum nigrum*, and *Wallemia sebi*. Our study shows that in patients with PCOS, the change in gut fungi is more significant than that in gut bacteria. According to Mims et al. [47], jejunal fungal communities are indeed dynamic and more susceptible to environmental influences than bacteria in healthy mice. The significance and value of gut fungi in the occurrence and development of PCOS are still not very clear, but previous studies may give some hints. For example, *Candida albicans* is considered as the major inducer of human antifungal Th17 cells [48] and increases interleukin-22 (IL-22) production [49]. Qi el at. [7] reported that *Bacteroides vulgatus* is markedly elevated in the gut microbiota and that the level of IL-22 is reduced. Taken together, both gut bacteria and gut fungi can affect the level of IL-22 and promote the occurrence and development of PCOS.At present, many hospitals and clinical centers use the Rotterdam standard for the diagnosis of PCOS. Haoula et al. [50] tentatively identified lipid biomarkers of PCOS, which may be useful in distinguishing PCOS according to targeted lipomics analysis. To find more effective and simple indicators used for the diagnosis and screening of PCOS, we built random forest models based on multiomics characteristics individually or their combination to discriminate PCOS from healthy controls. The findings showed that metabolites alone can achieve great performance in distinguishing disease from health, much better than microbiota-driven features, and that fungal features still perform better than bacterial genera in discriminating a PCOS status from a healthy status.

Our study still had limitations. Firstly, this is a single-center study with limited sample size, future multicenter research involving multiple different geographic areas are necessary to verify the data before clinical application. Secondly, although 16S rRNA gene sequencing analysis was conducted, higher taxa resolution until species level by metagenomics sequencing would enhance the results power. Thirdly, although multiomics data were used to reveal functional links between microbiome, metabolome and phenotype, cause-and-effect evidence which would address the chicken-and-egg debate problem need to be further clarified in animal studies. Last but not the least, fecal metabolomic analysis is also valuable to deeply understand how the gut microbiome metabolize directly.

In conclusion, integrated analysis of multiomics data from the gut bacteriome, mycobiome, metabolome and phenome showed that hyperandrogenemia plays a central role in the dysbiosis of intestinal microecology and the change in metabolic state in patients with PCOS and that its effect exceeds the role of BMI. Gut bacteria, gut fungi and their interactions may be important in the occurrence and development of PCOS. The priority of predictive models in discriminating PCOS status in this study were serum metabolites, fungal taxa and bacterial taxa.

## Supplementary Information

**Additional file 1:** **Table S1.** Clinical characteristics in healthy and PCOS group; **Table S2.** Sequence statistics and bacterial diversity indices from 16S rRNA and ITS2 gene sequencing; **Table S3.** The correlation between bacterial or fungal diversity and key clinical parameters; **Table S4.** The relative abundance comparison of bacterial genera among different groups; **Table S5.** The relative abundance comparison of fungal genera among different groups;** Table S6. **The relative abundance comparison of metabolites among different groups; **Table S7. **Mantel test between pairs of distance matrix

**Additional file 2:** **Figure S1.** Distribution of bacterial taxa at the phylum level and genus level; **Figure S2.** Reveal of characteristic bacterial taxa based on LDA Effect Size (LEfSe) analysis between Healthy-LB and PCOS-LB, between Healthy-HB and PCOS-HB; **Figure S3. **Distribution of fungal taxa at the phylum level and genus level;** Figure S4.** Reveal of characteristic fungal taxa based on LDA Effect Size (LEfSe) analysis between Healthy-LB and PCOS-LB; **Figure S5. **Reveal of characteristic fungal taxa based on LDA Effect Size (LEfSe) analysis between Healthy-HB and PCOS-HB; **Figure S6.** Serum metabolome changes in disease and healthy subjects; **Figure S7. **Reveal of characteristic metabolites based on LDA Effect Size (LEfSe) analysis between Healthy-LB and PCOS-LB; **Figure S8.** Reveal of characteristic metabolites based on LDA Effect Size (LEfSe) analysis between Healthy-HB and PCOS-HB; **Figure S9.** The bacterial genera and predicted pathway features were excavated on the base of Wilcox test comparison and Mean Decrease Gini by random forest importance parameter;** Figure S10.** The fungal genera and metabolites were excavated on the base of Wilcox test comparison and Mean Decrease Gini by random forest importance parameter; **Figure S11.** Correlation between bacterial diversity and fungal diversity.

## Data Availability

The 16S rRNA gene sequencing dataset supporting the conclusions of this article is available in the NCBI Sequence Read Archive repository under the accession number PRJNA786067 (https://submit.ncbi.nlm.nih.gov/subs/sra/SUB10762045/overview) and the ITS2 gene sequencing dataset is available in the SRA repository PRJNA786056 (https://submit.ncbi.nlm.nih.gov/subs/sra/SUB10756642/overview). The metabolome data is available in www.ebi.ac.uk/metabolights/MTBLS5982. Code and scripts used in the analyses are available upon request.

## Abbreviations

AD: Androstenedione
AFU: α-L-fucosidase
ALB: Albumin
ALP: Alkaline phosphatase
ALT: Alanine aminotransferase
AST: Aspartate aminotransferase
BMI: Body mass index
BUN: Urea nitrogen
CHE: Cholinesterase
Cr: Creatinine
DBIL: Direct bilirubin
DHEA: Dehydroepiandrosterone
DPP-IV: Dipeptidyl peptidase IV
E2: Estrogen
FSH: Follicle stimulating hormone
FAI: Free androgen index
FDR: False discovery rate
FKBP5-Met1: FKBP5 DNA methylation at CpG 35,657,180/hg19
FKBP5-Met2: FKBP5 DNA methylation at CpG 35,657,202/hg19
FKBP5-Met: Average of FKBP5 DNA methylation at CpG 35,657,180 and 35,657,202/hg19
G0: Glucose level at fasting status
G120: Glucose level at 120 min after glucose load
GGT: Glutamyltransferase
GLB: Globulin
HDL-C: High-density lipoprotein cholesterol
HOMA-IR: Homeostasis model assessment for insulin resistance
IBIL: Indirect bilirubin
I0: Insulin level at fasting status
I120: Insulin level at 120 min after glucose load
LDH: Lactate dehydrogenase
LDL-C: Low-density lipoprotein cholesterol
LH: Luteinizing hormone
LPC: Lysophosphatidyl choline
MAO: Monoamine oxidase
NAFLD: Nonalcoholic fatty liver disease
PCoA: Principal co-coordinates analysis
PCOS: Polycystic ovary syndrome
PRL: Prolactin
PROG: Progesterone
RBP-4: Retinol-binding protein 4
SHBG: Sex hormone-binding globulin
T2DM: Type 2 diabetes mellitus
TBIL: Total bilirubin
TC: Total cholesterol
TG: Triglycerides
TP: Total protein
TT: Total testosterone
UA: Uric acid
VIP: Variable importance in projection
