# Supplementary material for: Alterations of bacteriome, mycobiome and metabolome characteristics in PCOS patients with normal/overweight individuals
Source: J Ovarian Res. 2022 Oct 28;15:117. doi: 10.1186/s13048-022-01051-8 (PMC9613448; doi:10.1186/s13048-022-01051-8)

**Figure S1** Distribution of bacterial taxa at the phylum level **(a)** and genus level **(b)**. The 20 genera of top relative abundance were chose.


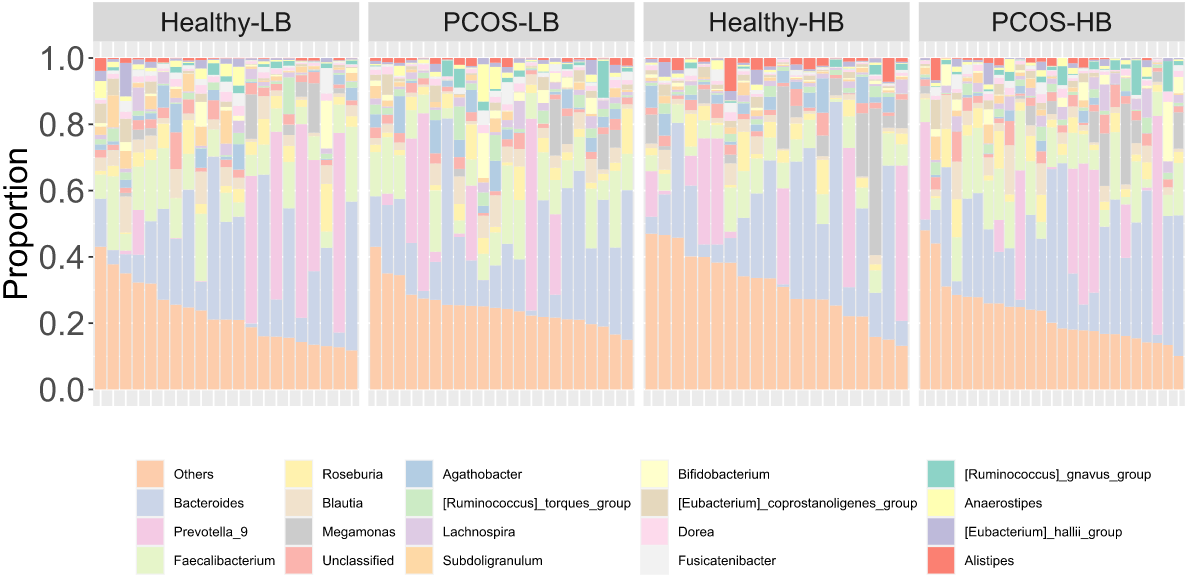

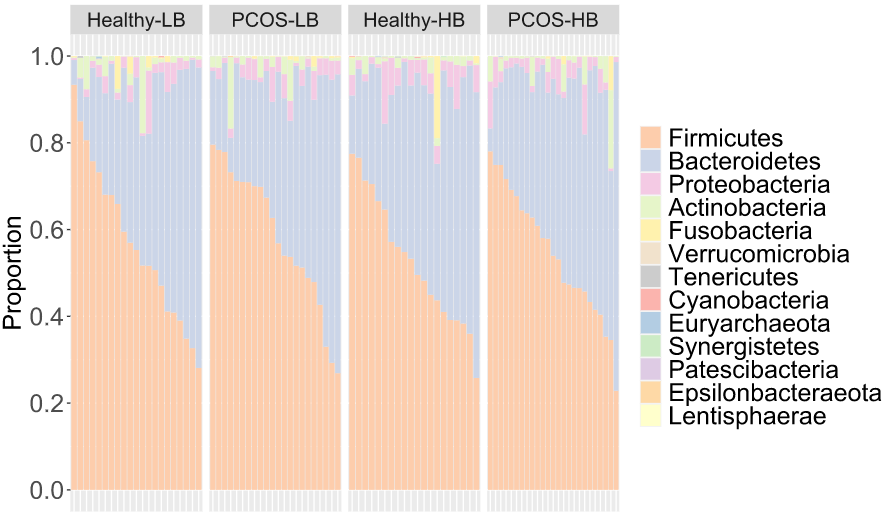


a

b

**Figure S2** Reveal of characteristic bacterial taxa based on LDA Effect Size (LEfSe) analysis.**(a)** Comparison between Healthy-LB and PCOS-LB. **(b)** Comparison between Healthy-HB and PCOS-HB.

a


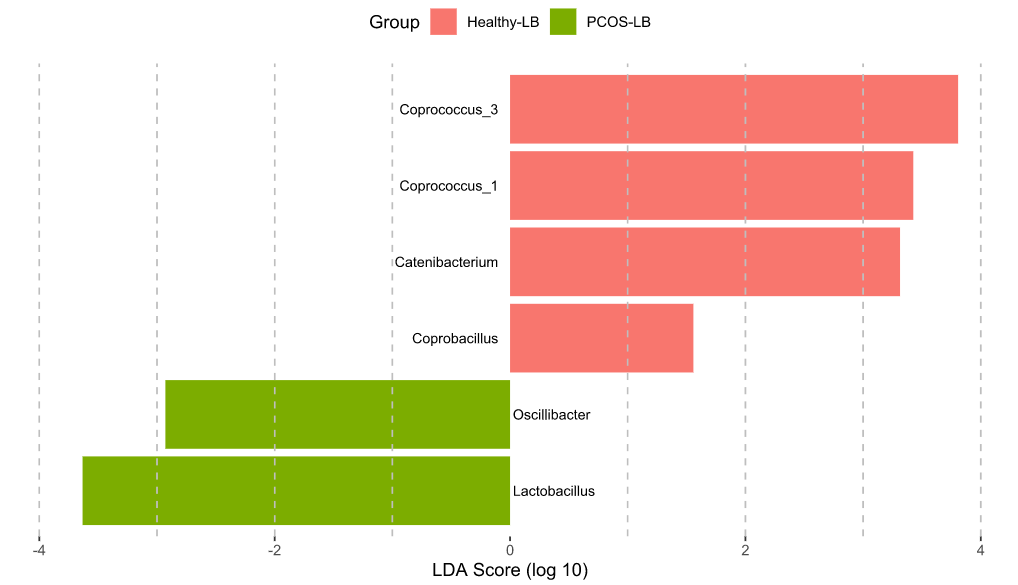


b


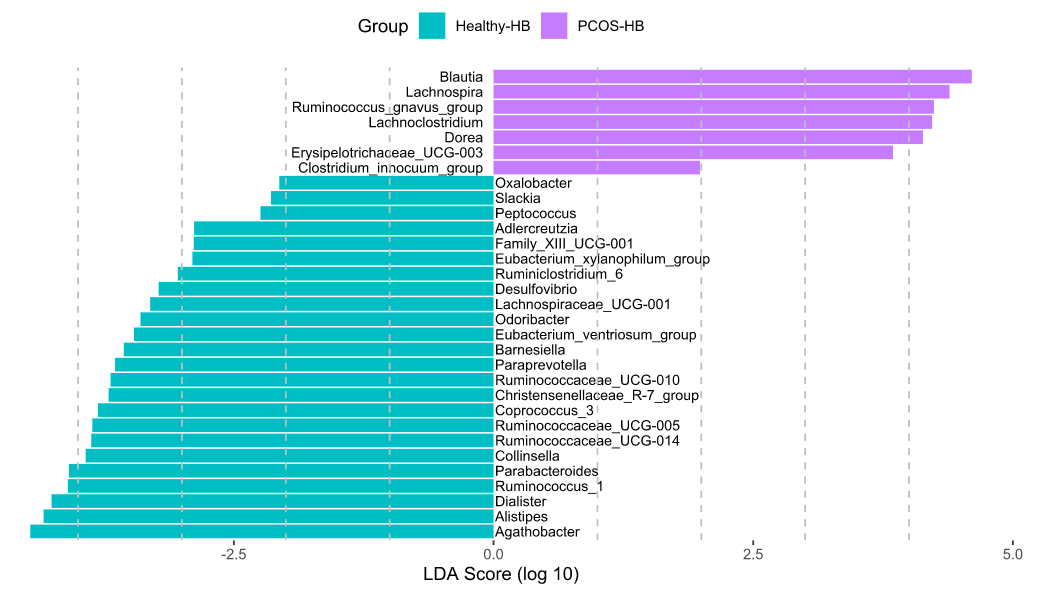


**Figure S3** Distribution of fungal taxa at the phylum level **(a)** and genus level **(b)**. The 20 genera of top relative abundance were chose.


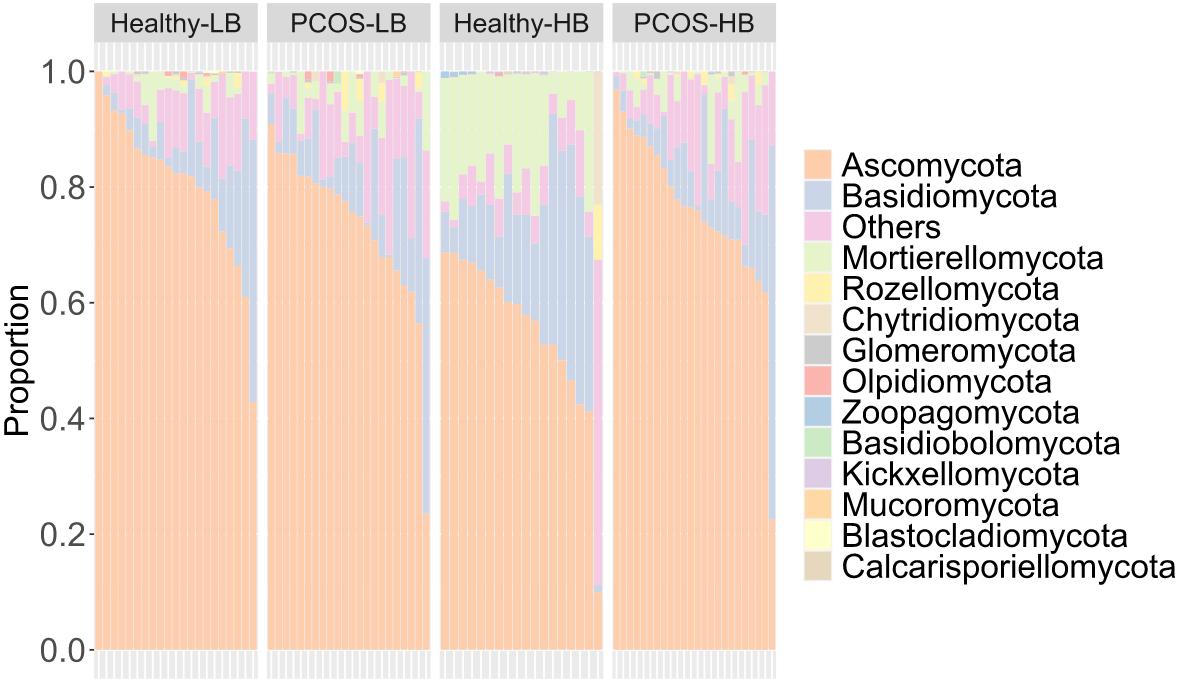

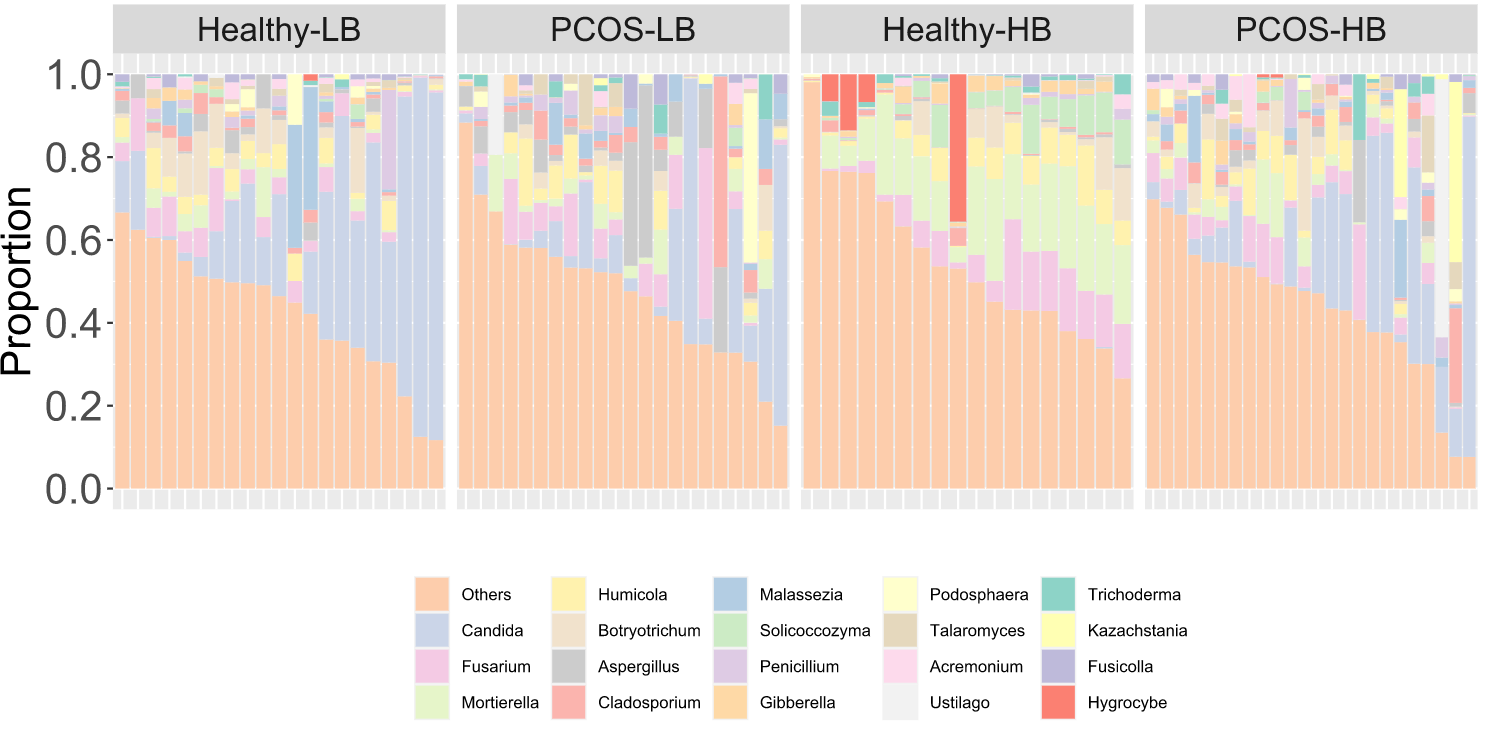


a

b

**Figure S4** Reveal of characteristic fungal taxa based on LDA Effect Size (LEfSe) analysis. Comparison between Healthy-LB and PCOS-LB.


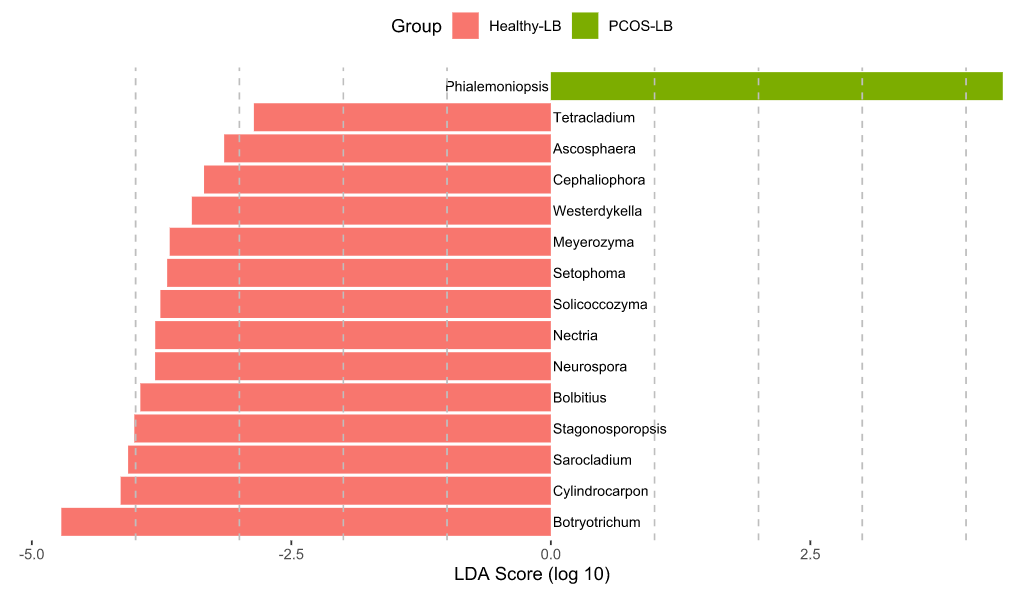


**Figure S5** Reveal of characteristic fungal taxa based on LDA Effect Size (LEfSe) analysis. Comparison between Healthy-HB and PCOS-HB.


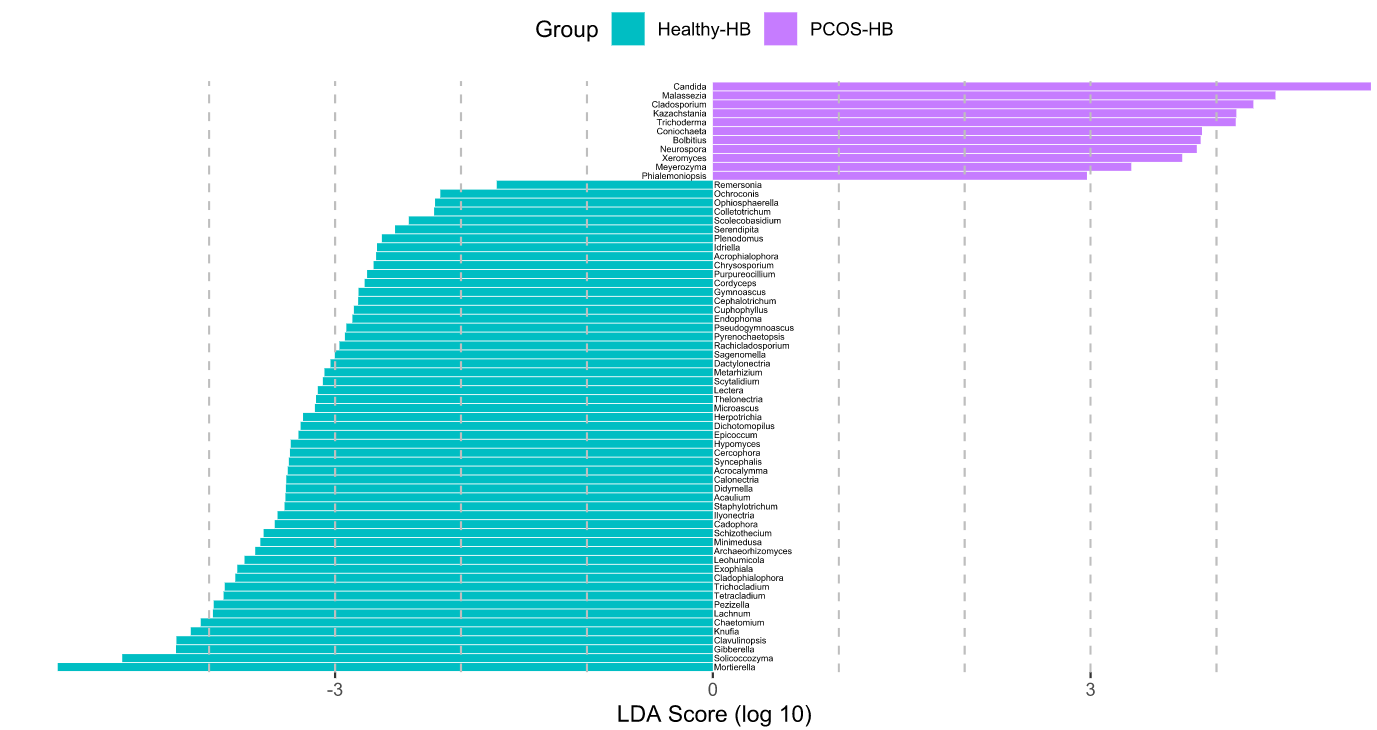


**Figure S6 Serum metabolome changes in disease and healthy subjects**. **(a)** RDA analyses reflecting differences in gut metabolites structures fitted with significantly correlated clinical properties. **(b)** Volcano plot demonstrated metabolites change in PCOS-LB, compared with Healthy-LB. **(c)** Volcano plot demonstrated metabolites change in PCOS-HB, compared with Healthy-HB. The X axis indicates log2-transformed FC of serum metabolites abundance and Y axis denotes FDR adjusted *p* value analysed using Wilcox test. **(d)** The correlation heatmap showing the interaction between key clinical parameters and characterized top 40 metabolites.


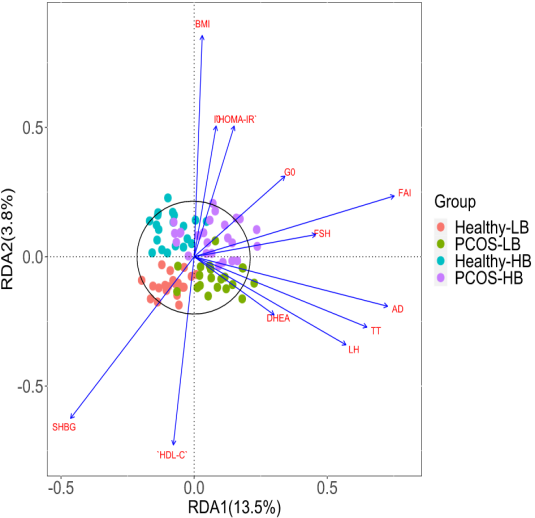

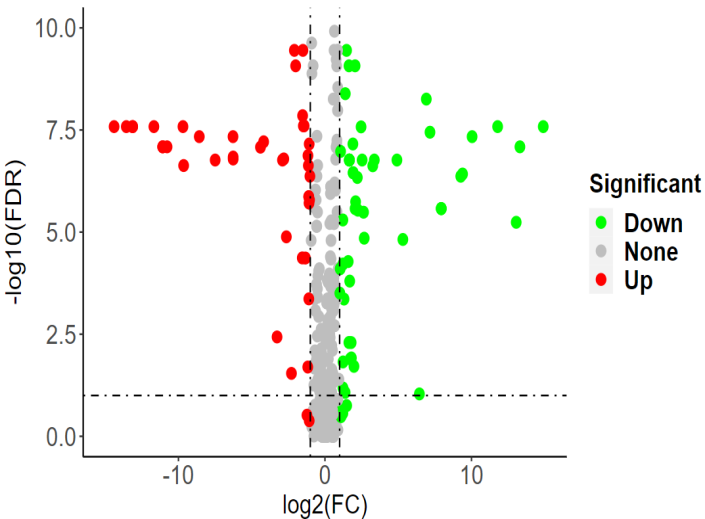

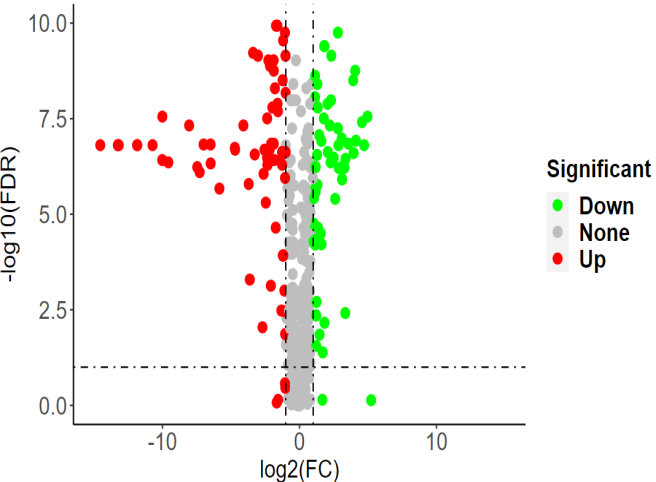

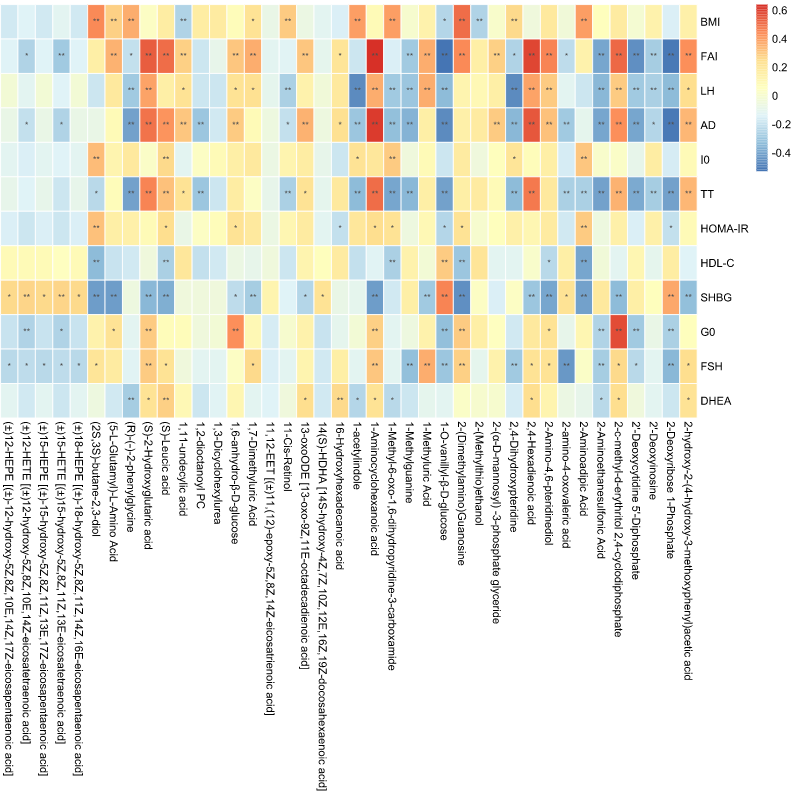


a

b

c

d

**Figure S7** Reveal of characteristic metabolites based on LDA Effect Size (LEfSe) analysis. Comparison between Healthy-LB and PCOS-LB.


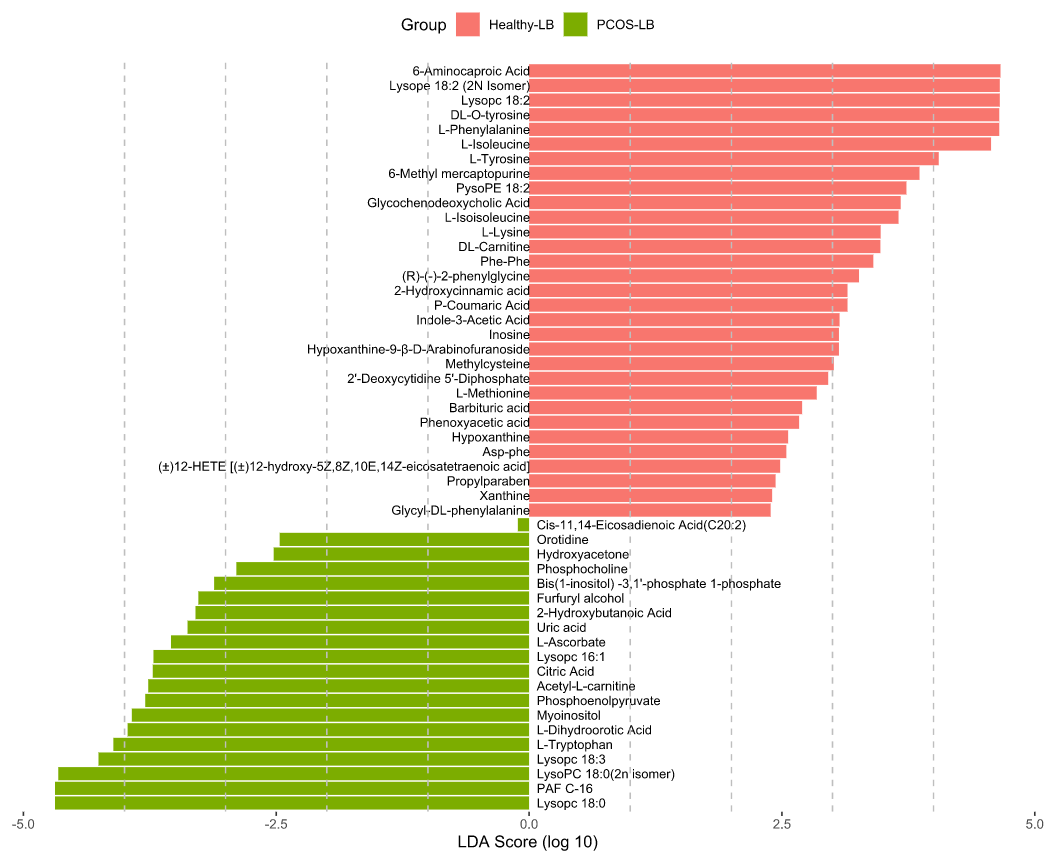


**Figure S8** Reveal of characteristic metabolites based on LDA Effect Size (LEfSe) analysis. Comparison between Healthy-HB and PCOS-HB.


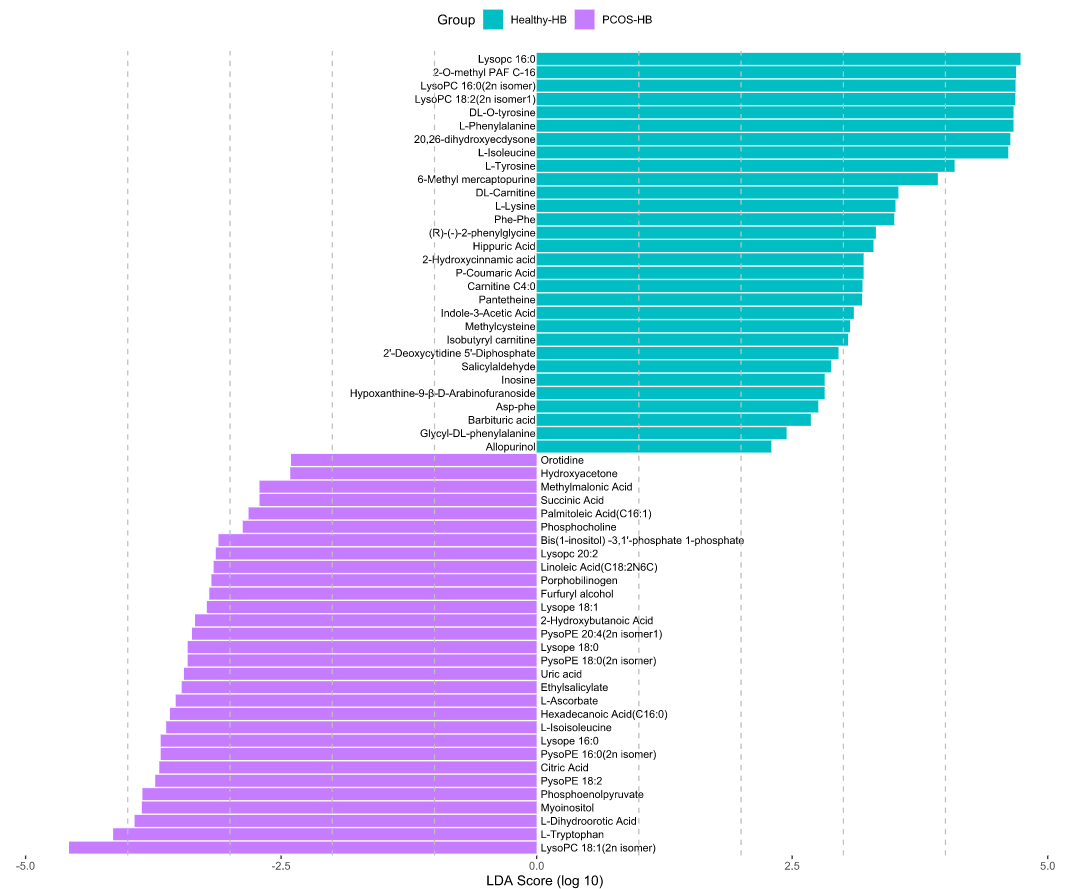


**Figure S9** The bacterial genera and predicted pathway features were excavated on the base of Wilcox test comparison and Mean Decrease Gini by random forest importance parameter. **(a,b,c)** Bacterial genera used for disease diagnose with comparison between the pairs of PCOS vs Healthy, PCOS-LB vs Healthy-LB, PCOS-HB vs Healthy-HB. **(d,e,f)** Pathway used for disease diagnose with comparison between the pairs of PCOS vs Healthy, PCOS-LB vs Healthy-LB, PCOS-HB vs Healthy-HB.


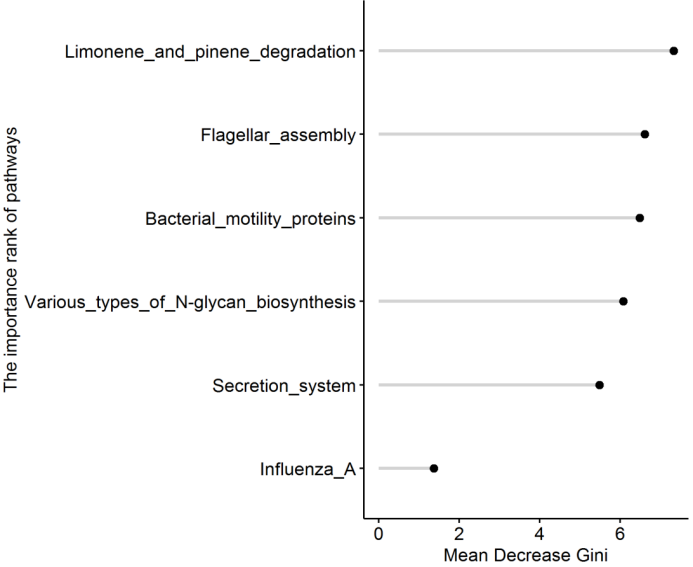

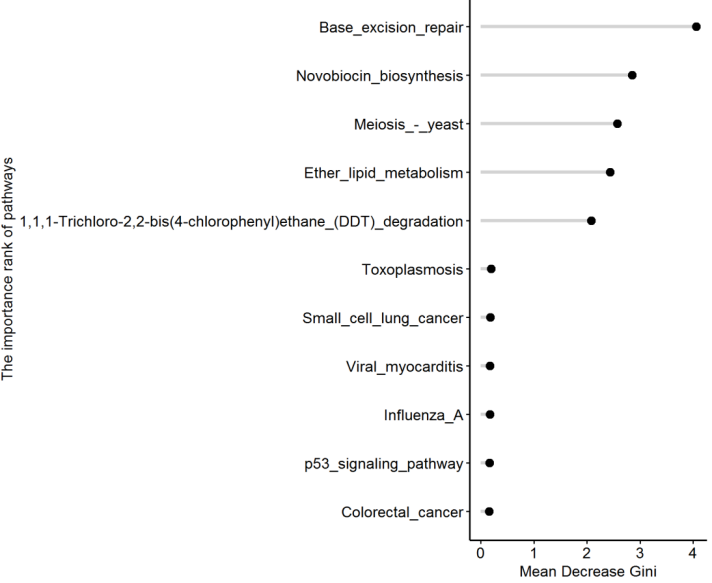

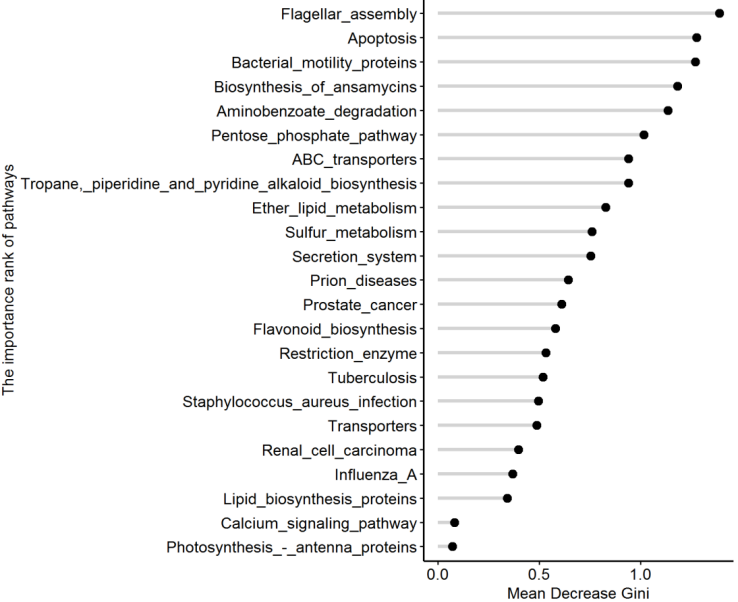


d

e

f


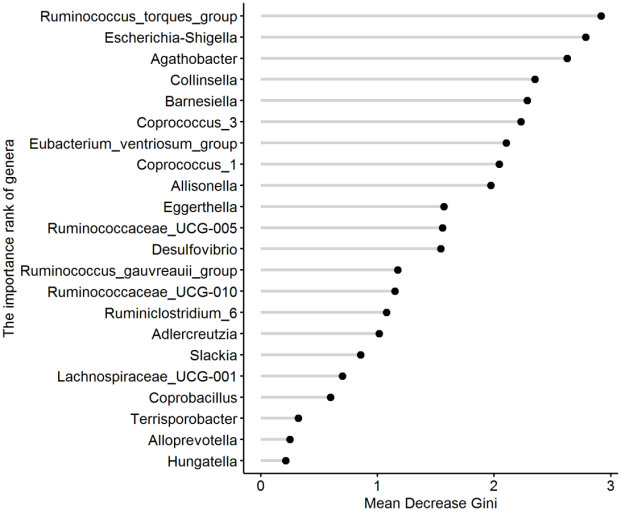

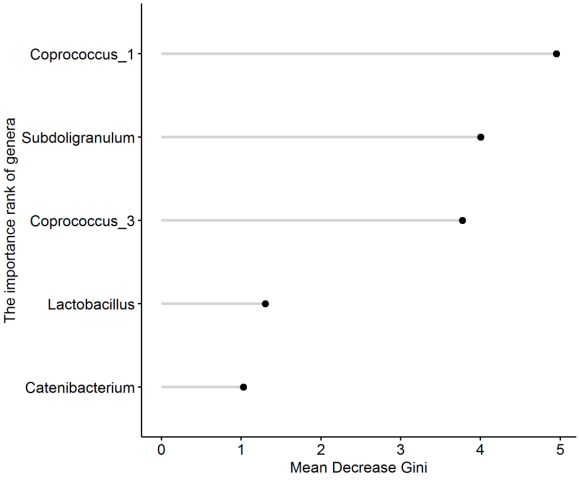

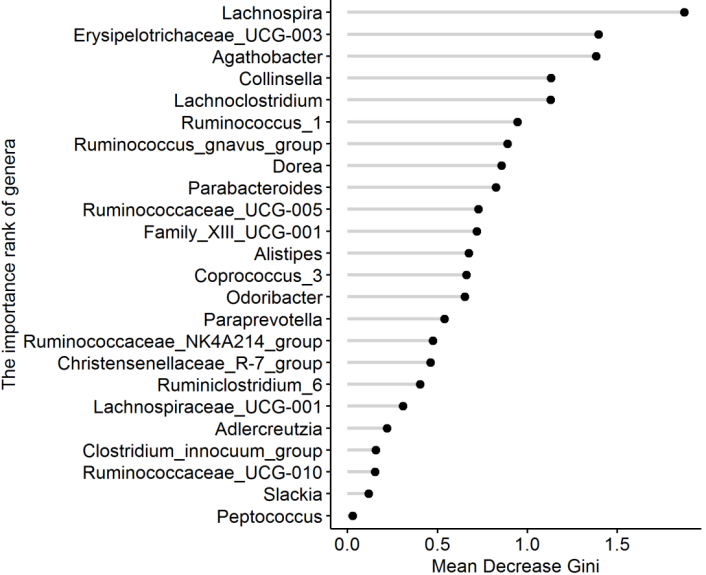


a

b

c

**Figure S10** The fungal genera and metabolites were excavated on the base of Wilcox test comparison and Mean Decrease Gini by random forest importance parameter. **(a,b,c)** Fungal genera used for disease diagnose with comparison between the pairs of PCOS vs Healthy, PCOS-LB vs Healthy-LB, PCOS-HB vs Healthy-HB. **(d,e,f)** Metabolites used for disease diagnose with comparison between the pairs of PCOS vs Healthy, PCOS-LB vs Healthy-LB, PCOS-HB vs Healthy-HB.


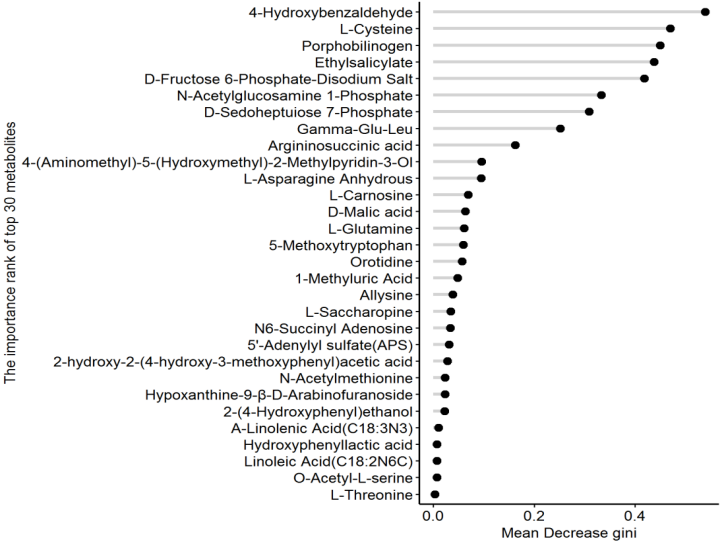

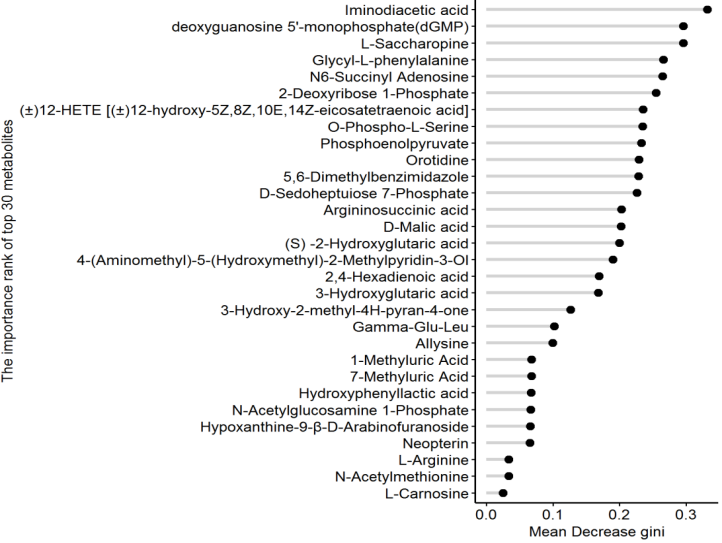

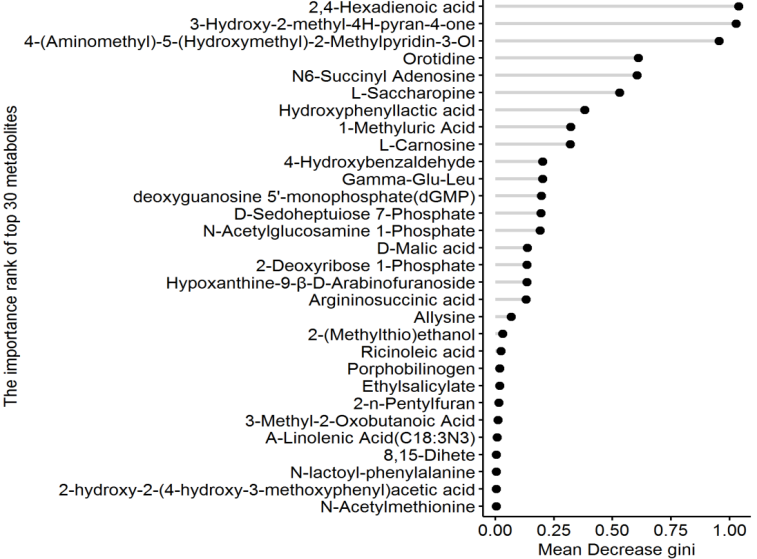

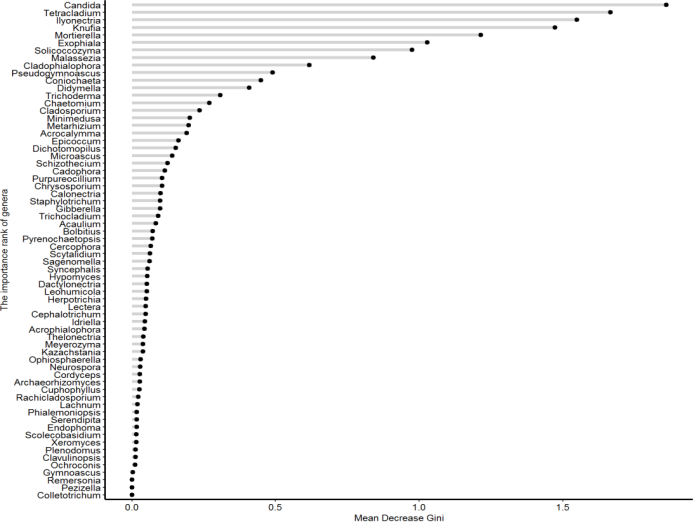

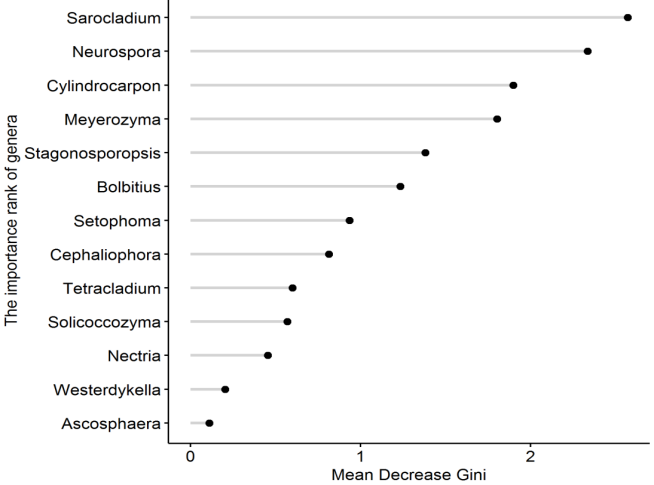

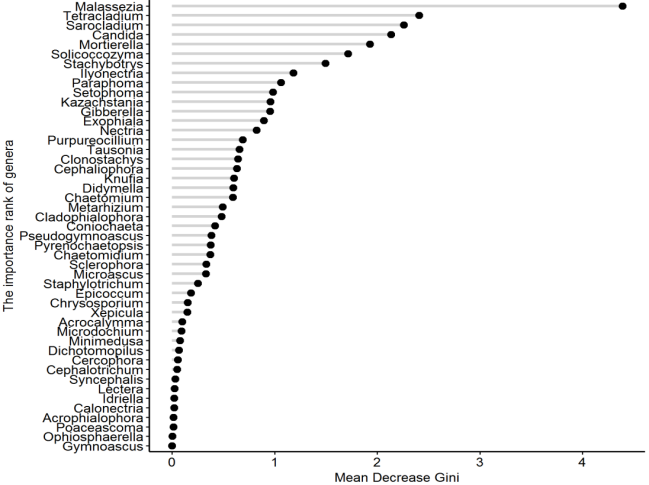


a

b

c

d

e

f

**Figure S11** Correlation between bacterial diversity and fungal diversity. **(a)** The linear relationship between bacterial Chao1 and fungal Chao1 index. **(b)** The linear relationship between bacterial Shannon and fungal Shannon index.


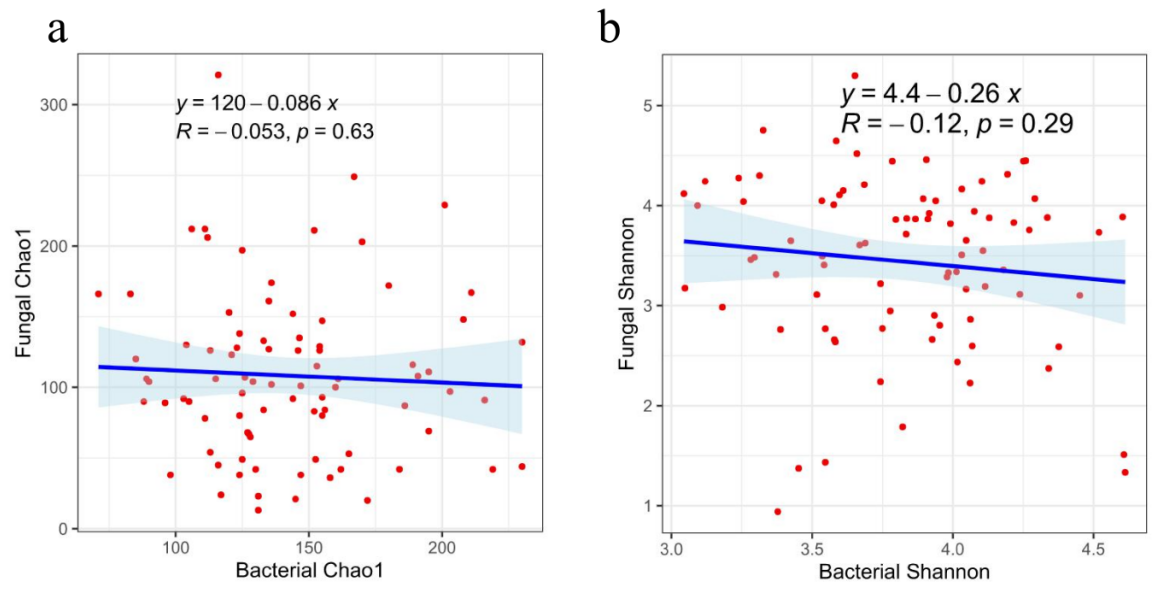

Supplement: Supplementary file 2 — Additional file 2: Figure S1. Distribution of bacterial taxa at the phylum level and genus level; Figure S2. Reveal of characteristic bacterial taxa based on LDA Effect Size (LEfSe) analysis between Healthy-LB and PCOS-LB, between Healthy-HB and PCOS-HB; Figure S3. Distribution of fungal taxa at the phylum level and genus level; Figure S4. Reveal of characteristic fungal taxa based on LDA Effect Size (LEfSe) analysis between Healthy-LB and PCOS-LB; Figure S5. Reveal of characteristic fungal taxa based on LDA Effect Size (LEfSe) analysis between Healthy-HB and PCOS-HB; Figure S6. Serum metabolome changes in disease and healthy subjects; Figure S7. Reveal of characteristic metabolites based on LDA Effect Size (LEfSe) analysis between Healthy-LB and PCOS-LB; Figure S8. Reveal of characteristic metabolites based on LDA Effect Size (LEfSe) analysis between Healthy-HB and PCOS-HB; Figure S9. The bacterial genera and predicted pathway features were excavated on the base of Wilcox test comparison and Mean Decrease Gini by random forest importance parameter; Figure S10. The fungal genera and metabolites were excavated on the base of Wilcox test comparison and Mean Decrease Gini by random forest importance parameter; Figure S11. Correlation between bacterial diversity and fungal diversity. [file 13048_2022_1051_MOESM2_ESM.docx]
